# The Exchange of Cyclometalated Ligands

**DOI:** 10.3390/molecules26010210

**Published:** 2021-01-03

**Authors:** Alexander D. Ryabov

**Affiliations:** Institute for Green Science, Department of Chemistry, Carnegie Mellon University, 4400 Fifth Avenue, Pittsburgh, PA 15213, USA; ryabov@andrew.cmu.edu

**Keywords:** exchange of cyclometalated ligands, cyclometalation, transcyclometalation, C–H bond activation, palladium, platinum, ruthenium, manganese, rhodium, iridium, reaction mechanism

## Abstract

Reactions of cyclometalated compounds are numerous. This account is focused on one of such reactions, the exchange of cyclometalated ligands, a reaction between a cyclometalated compound and an incoming ligand that replaces a previously cyclometalated ligand to form a new metalacycle: 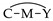 + H-C*~Z ⇄ 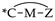 + H-C~Y. Originally discovered for Pd^II^ complexes with Y/Z = N, P, S, the exchange appeared to be a mechanistically challenging, simple, and convenient routine for the synthesis of cyclopalladated complexes. Over four decades it was expanded to cyclometalated derivatives of platinum, ruthenium, manganese, rhodium, and iridium. The exchange, which is also questionably referred to as transcyclometalation, offers attractive synthetic possibilities and assists in disclosing key mechanistic pathways associated with the C–H bond activation by transition metal complexes and C–M bond cleavage. Both synthetic and mechanistic aspects of the exchange are reviewed and discussed.

## Contents

1. Introduction
2. The Exchange Discovery
3. Scope: A Neat Synthetic Procedure
4. Cyclopalladation of Phosphorous and Antimony Donor Ligands Including *o*-Carboranes
5. Asymmetric Version of the Ligand Exchange
6. The Mechanism of Palladium *N*/*N* Ligand Exchange
*6.1. Thermodynamics of Palladium(II) Exchange*
*6.2. Kinetics and Mechanism of Palladium(II) Exchange*
*6.3. “Mechanism” of Thermodynamic Control*
*6.4. A Mechanistic Comment Regarding Section 5*
7. Processes Mechanistically Relevant to Palladium Ligand Exchange
*Palladation by η^3^-Allyl Palladium(II) Dimers*
8. Exchange in the Platinum Series
*8.1. η^3^-Allyls as Leaving Ligands*
*8.2. True Exchange of Cycloplatinated Ligands*
*8.3. Intramolecular Exchange of Cycloplatinated Ligands*
*8.4. Pincer-Pincer Ligand Exchange in Cycloplatinated Complexes*
*8.5. Exchange of Cyclometalated Ligands or Transcyclometalation?*
9. The Ligand Exchange with Pincer Ruthenium Complexes
10. Other Examples of Exchange of Cyclometalated Ligands
*10.1. Manganese(I)*
*10.2. Rhodium(III)*
*10.3. Iridium(III)*
11. Summary and Conclusions

## 1. Introduction

I am not sure that I would start writing this account under any circumstances other than for a Special Issue of *Molecules* dedicated to Dr. Michel Pfeffer, my colleague, partner, coworker and, let me hope, a good friend. Michel played a huge role in my life, contributing to its both scientific and non-scientific segments. His earlier brilliant works and a review on cyclopalladation [1] were stimulative, inspirational, and highly contagious. As a result, cyclopalladation and reactions of cyclopalladated compounds became my early research priority. Our first meeting at a conference in Sardinia in 1992 launched a long-term fruitful collaboration and I am very proud of the results we achieved together. My four visits to Strasbourg and time spent with Michel were all so scientifically and culturally rewarding that I regret deeply that this happened last time in October of 2012. My research career flows to the end and I state with confidence that Michel appeared to be my best superior collaborator, the easiest scientist to work with. Perhaps his words that “a collaboration goes perfect when you trust your partners 100%” could be applied to me? My trust in Michel was always much above 100%. 

**Scheme 1:**
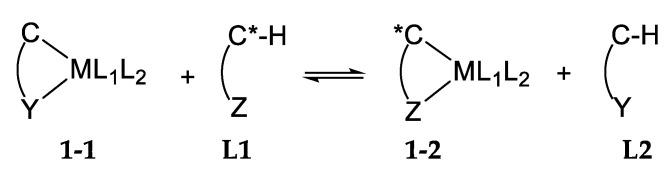
Exchange of cyclometalated ligands involving a cyclometalated compound and an incoming ligand that replaces a previously cyclometalated ligand to form a new metalacycle.

There were no doubts that a contribution to a Special Issue, the “Michel’s” Issue, had to be centered around cyclometalation and reactions of cyclometalated compounds just to highlight his immeasurable contribution to this field of inorganic/organometallic chemistry. I decided to review reactions of exchange of cyclometalated ligands as they are presented in Scheme 1. A cyclometalated compound like **1-1** reacts with an incoming ligand **L1**, the C–H bond of which can also be metalated by a metal center M. As a result, **L1** replaces the previously cyclometalated ligand of **1-1** to form a new cyclometalated complex **1-2**. 

Mechanistically challenging reaction in Scheme 1, which involves C–M bond breaking and C*–H bond activation, was first described for palladium(II) complexes but was further expanded, inter alia, at platinum(II) and rhodium(III) centers, i.e., at the metal centers with options for C–H bond cleavage via electrophilic substitution pathways [2]. The C–M bond breaking is performed as protonolysis and therefore acidic components/cosolvents should usually be present in reaction mixtures such as acetic and trifluoroacetic acids or tiny amounts of much stronger acids.

## 2. The Exchange Discovery

**Scheme 2:**
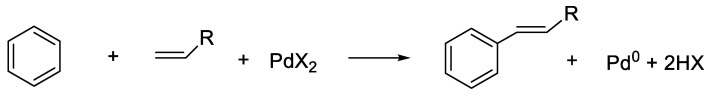
Fujiwara–Moritani arylation of alkenes by arenes.

**Scheme 3:**
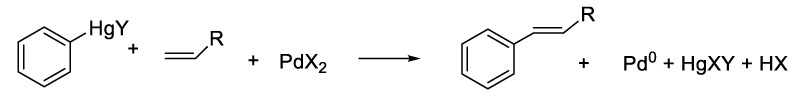
Heck arylation of alkenes by organomercurials.

Our kinetic and mechanistic studies of the palladium(II)-promoted Fujiwara–Moritani arylation of alkenes by arenes (Scheme 2) and its organometallic Heck version (Scheme 3) [3] confirmed that the rate-limiting step is the formation of an aryl-palladium intermediate in both cases, which then reacts rapidly with alkenes. Thus, no kinetic and mechanistic information could be acquired for the insertion of alkenes into Pd–C bonds. Cyclopalladated complexes such as **1-1** or **1-2** contained “pre-formed” Pd–C bonds and looked like convenient starting materials for mechanistic studies of the alkene insertion.

**Scheme 4:**
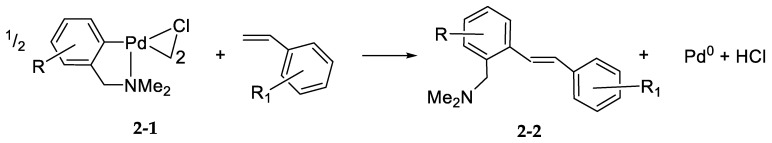
Arylation of alkenes by palladacycles.

Chloro-bridged orthopalladated *N,N*-dimethylbenzylamine dimers **2-1** were selected as starting materials for studies of reactions with *para*-substituted styrenes (Scheme 4) [4] because the ability of the former to react with alkenes was already well documented [5]. The reaction run in acetic acid solvent appeared mechanistically remarkable for several reasons [6]. First, its reactants **2-1** and styrenes behaved both as nucleophiles with respect to each other, i.e., both electron-donating groups R and R_1_ increased the rate of alkene insertion into the Pd–C bond. Second, neutral salts such as NaClO_4_ unprecedently accelerated reaction in Scheme 4 due to the specific acid catalysis which manifested because of the equilibrium HOAc + NaClO_4_ ⇄ HClO_4_ + NaOAc and tiny concentrations of HClO_4_ did in fact accelerate the reaction. 

**Figure 1:**
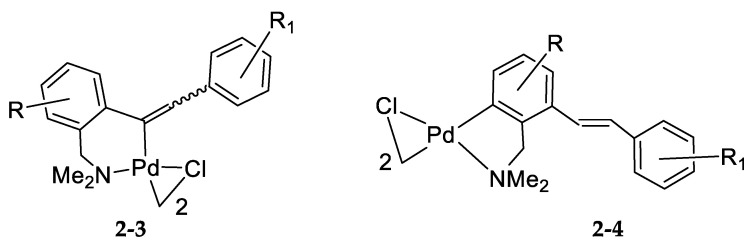
Structures of **2-3** and **2-4**.

**Scheme 5:**
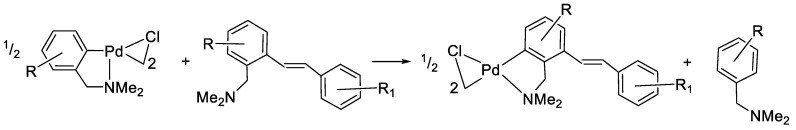
The exchange between starting material **2-1** and reaction product **2-2**.

The most prominent fact was the isolation of a palladium(II)-containing derivative of **2-2** under the synthetic conditions [4]. There were no doubts that the material was a palladacycle and it was hypothesized that the alkene *sp*^2^ C–H bond underwent metalation to form a six-membered ring within **2-3** (Figure 1) [4]. Detailed examination of the product composition revealed that in fact the aromatic C–H bond was palladated and complex **2-4** was formed [7]. The question that remained was *how* because zero-valent palladium is produced in reaction in Scheme 4 but the C–H bond activation via oxidative addition involving palladium(0) did not look to be an acceptable pathway [2]. It turned out that **2-4** resulted from the exchange process between starting material **2-1** and reaction product **2-2** (Scheme 5) because the alkene insertion into the Pd–C bond was run in acetic acid as a solvent or co-solvent [7]. The reaction in Scheme 5 was the first example of what later was referred to as the exchange of cyclometalated ligands.

## 3. Scope: A Neat Synthetic Procedure

The ligand exchange is an attractive synthetic method for an easy preparation of chloro- and acetato-bridged palladacycles with an *N*-donor center within a metalacycle (Table 1). Convenient starting materials are dimeric chloro- and acetato-bridged cyclometalated derivatives of *N,N*-dimethylbenzylamine (**3-1** and **3-2**, respectively) or dimethylaminomethyferrocene (**3-3**). The reactions are run for several hours or overnight under rather mild conditions in chloroform, benzene or toluene mixed with an organic acid cosolvent. The chloro version of the exchange affords, as a rule, beautiful crystalline products in good yields (Table 1), which are needed just to filter off without additional purification. Michel Pfeffer and co-workers extended the exchange at an *S*-containing ligand (entries 12–14 in Table 1) and *S*-palladacycles were isolated in excellent yields.

Acetato-bridged dimeric palladacycles are more soluble in mixed organic solvents and therefore the products did not always crash out from reaction solutions. However, their use is advantageous in the case of incoming ligands, which are less stable in acidic media and may undergo solvolysis. For example, benzylidene aniline reacts cleanly with acetato-bridged **3-2** to afford the corresponding palladacycle (entry 9 in Table 1) [9], whereas the reaction in the case of the chloro-bridged analogue **3-1** was complicated by the solvolysis of benzylidene aniline, which gave the [PdCl_2_(PhNH_2_)_2_] byproduct. 

**Scheme 6:**
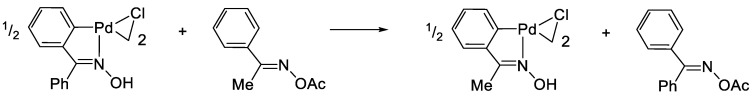
The exchange with *O*-acetyl oxime.

The ligand exchange is convenient for preparation of various cyclopalladated oximes [13,16,17] which find various applications in catalysis [18]. Benzaldehyde, acetophenone, propiophenone, and benzophenone oximes react cleanly with **3-1** (R = H) to form palladacycles **3-6** in high yields [13]. Ferrocene-based and benzophenone oxime palladacycles (**3-3** and **3-6**, respectively) can be used instead of **3-1** with the same incoming oximes and the products are formed in 76–92% yields. Cyclopalladated aryl oxime **3-7** with a 15-crown-5 motif was prepared in a 72% yield from oxime of 4’-acetylbenzo-15-crown-5 and **3-1** (entry 17 in Table 1). Its solubility in water is more than ten times higher than that of the related complex without the crown fragment and increases further in the presence of Mg^II^ salts [16]. The exchange with oxime ligands offered a curious reaction given by Scheme 6 when an attempt was made to obtain the *O*-acetyl oxime chloro-bridged dimers starting, for example, from acetophenone *O*-acetyl oxime and the corresponding cyclopalladated chloro-bridged dimers. No reaction was observed using **3-1** or **3-3**. With complex **3-6** (R = Ph) as a palladating agent, the reaction does occur (Scheme 6), but the products are complex **3-6** (R = Me) and benzophenone *O*-acetyl oxime. The reaction is formally the exchange of phenyl and methyl groups, but it surely does not involve C–C bond metathesis. The mechanism probably involves the ligand exchange to produce *ortho*-palladated acetophenone *O*-acetyl oxime and free benzophenone oxime. The latter, a powerful nucleophile, deacylates the *ortho*-palladated product to afford free *O*-acylated benzophenone oxime. Scheme 6 accounts for the unsuccessful attempts to prepare *ortho*-palladated *O*-acetyl oximes by reacting them with Na_2_PdCl_4_ in MeOH as solvent [19]. After the formation, the product loses the acyl function as a result of the nucleophilic attack by methanol.

2-Phenyl-2-oxazoline is cleanly cyclopalladated by **3-1** (entry 16 in Table 1) [15]. In toluene, in the absence of acetic acid, there is just a cleavage of chloro-bridges in **3-1** by the oxazoline to form the monomeric complex. Interestingly, even in the presence of HOAc but at a lower temperature, i.e., under ambient conditions, only the *N*-bound co-ordination complex [PdCl_2_L_2_] was formed, and attempts failed to convert it to the cyclopalladated dimer by heating in an HOAc–CHCl_3_ mixture [15].

Table 1 is far from being complete though it provides a solid picture of a synthetic potential of the exchange of cyclopalladated ligands. Many more examples were described by other workers who, inter alia, focused on the formation of five- and six-membered palladacycles with *N*-donor atom, analyzed approaches to the formation of Pd–C_sp2_ and Pd–C_sp3_ bonds and as well as to exo- and endo-palladacycles in the case of polyfunctional, usually imine, ligands [20,21,22,23,24]. Granell et al. explored complexes **3-5** (R = Cl and NMe_2_) as a palladium(II) source [11]. The latter complex was found to be unstable under the reaction conditions. It is also worth stating that the exchange of cyclopalladated ligands may occur without solvent. Silica gel can be successfully used as a ‘‘green’’ medium for this reaction. Cyclopalladation on SiO_2_ required an equimolar amount of CF_3_CO_2_H. Good yields of the Pd^II^ transfer from **3-1** and **3-2** at benzyl methyl sulfide, benzyl phenyl sulfide, benzyldiphenylphosphine, 8-methylquinoline and 1,3-bis(methylthiomethyl)benzene were obtained at higher temperatures (80–100 °C) and longer reaction times (ca. 24 h except for the phosphine) at efficient stirring [25].

## 4. Cyclopalladation of Phosphorous and Antimony Donor Ligands Including *o*-Carboranes

**Figure 2:**
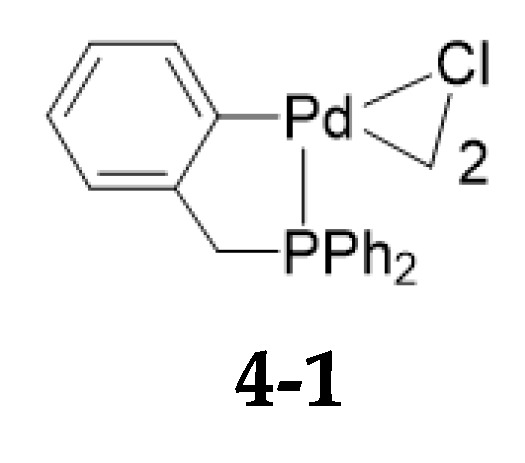
Structure of **4-1**.

A prelude to the synthesis of a variety of cyclopalladated *o*-carboranes via the ligand exchange was the preparation of **4-1** (Figure 2) reacting *N*-donor palladacycle **3-1** with benzyldiphenylphosphine PhCH_2_PPh_2_ [26]. Though the isolated yield was low (21%), it was still higher than that achieved by metalation of PhCH_2_PPh_2_ by Pd^II^ acetate [27]. This result stimulated studies with a series of *o*-carborane derivatives with P and As donor atoms as incoming ligands **L1** in Scheme 1 though with a noticeable variation because B–H bonds had to be cleaved instead of C–H bonds as in all cases described above so far.

**Figure 3:**
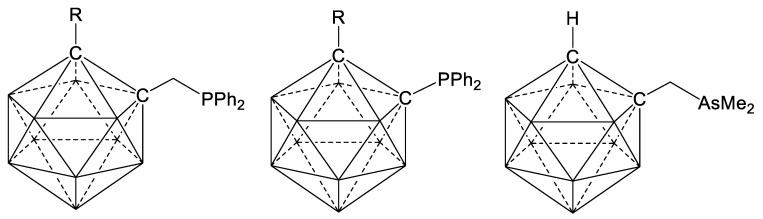
*P*- and *As*-donor ligands derived from *o*-carborane.

Palladium(II) has been successfully transferred via the ligand exchange from dimeric chloro-bridged *N*-donor palladacycles **3-1** and both enantiomers of **3-4** (R = Me) at several *P*- and *As*-donor ligands **4-2**–**4-4** (Figure 3) derived from the parent *o*-carborane C_2_B_10_H_12_ [26].

**Figure 4:**
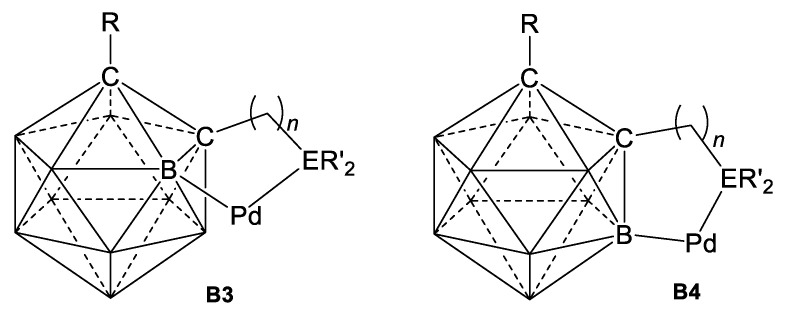
Two possible isomers of the cyclopalladated *o*-carborane derivatives.

Dimers **4-5** are effortlessly prepared in high yields. In particular, **4-2a** reacts with an equimolar amount of **3-1** at 70 °C in a HOAc-CHCl_3_ mixture to afford five-membered palladacycle **4-7** in an 83% yield. The product from **4-2b** was isolated in a 95% yield. Both products are mixtures of B3 (major) and B4 (minor) palladated species (Figure 4). The B3/B4 ratio is sterically determined by the nature of R (100% for B4 in the case of **4-3c** (R = *^i^*Pr).

The “carborane version” of the exchange reaction is limited by ligands with phosphorus donor atoms. Ligands with *N*-donor arms −CH_2_NEt_2_ and −N=NPh reduce palladium(II) in acetic acid medium, as carboranes without hanging donor centers react with Pd^II^ acetate to produce boric acid [28]. 

**Scheme 7:**
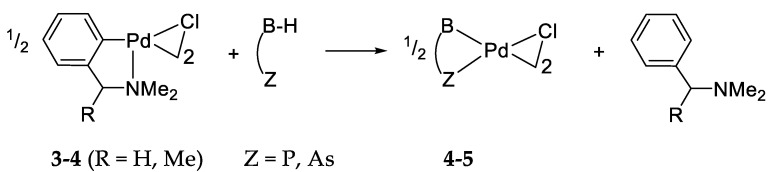
Reactions with *R* and *S* enantiomers of **3-4**.

The interaction between **3-1** and **4-2a** begins with the cleavage of chloro-bridges of the palladium dimer by the phosphorus donor. No such cleavage occurs during the formation of the four-membered palladacycles, see below. Since mono-substituted *o*-carborane is prochiral [29], the reaction with *R* and *S* enantiomers of **3-4** was investigated (Scheme 7) to reveal that the product was racemic in both instances consistent with a dissociative route of the exchange (see below). A five-membered palladacycle with an As donor atom derived from **4-4** was also obtained via the ligand exchange. Four-membered cyclopalladated carboranes were also be prepared in the same way but even under milder conditions. In particular, **4-3c** and **3-1** react in a HOAc-CHCl_3_ mixture at room temperature to give corresponding **4-6** in an isolated 40% yield. No pre-equilibrium bridge-splitting by **4-3** takes place, apparently due to their large cone angles. Isopropyl C–H bonds of **4-3c** are not metalated. Contrary to five-membered palladacycles from **4-2**, the ^11^B NMR spectroscopy of four-membered species containing **4-3** proved the Pd–B bond formation. 

**Scheme 8:**
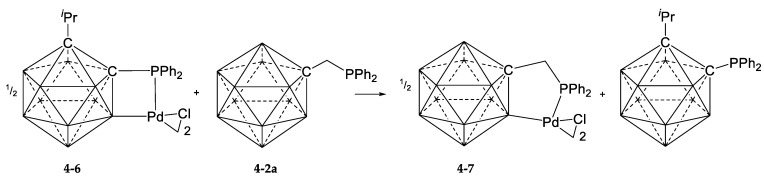
The ligand exchange involving a *P*-donor *B*-cyclopalladated complex and a *P*-donor incoming ligand.

The transformations in the carborane series offered an example of the ligand exchange involving a *P*-donor *B*-cyclopalladated complex and a *P*-donor incoming ligand, the B–H bond of which is metalated (Scheme 8). The reaction between the four-membered cyclopalladated chloro-bridged dimer **4-6** and free **4-2a** yields the five-membered *B*-palladated complex **4-7** in 65% yield. The *P*/*P* exchange takes place when just **4-2a** is the incoming ligand. No similar migration of Pd^II^ was observed at PhCH_2_PPh_2_ or *o*-PhCB_10_H_10_CN=NPh.

## 5. Asymmetric Version of the Ligand Exchange

Chiral cyclopalladated complexes represent a rather advanced area of organometallic chemistry. Significant progress was reached due to pioneering studies of Russian organometallic chemists, among which Sokolov [30,31] and Dunina [32] should be mentioned first. Asymmetric induction during the cyclopalladation of C–H bonds of prochiral ligands is known since 1979 [33]. Therefore, it was challenging to explore asymmetric options of reaction in Scheme 1 using optically active palladium(II) complexes as starting materials. First attempts to transfer chirality from cyclopalladated complexes **3-4** of *R*- and *S-N,N*-dimethyl-1-phenylethan-1-amine at the methylene group of 8-ethylquinoline (entry 6 in Table 1) were unsuccessful though yields of the cyclopalladated product were satisfactory [10]. This fact seemed as anticipated in terms of the dissociative mechanism of the palladium ligand exchange involving *N*-donor palladacycle and *N*-donor incoming ligand in acidic media (see Section 6) and therefore further studies in this direction were put on hold until Dunina and co-workers disclosed the asymmetric potential of a palladium version of reaction in Scheme 1.

**Scheme 9:**
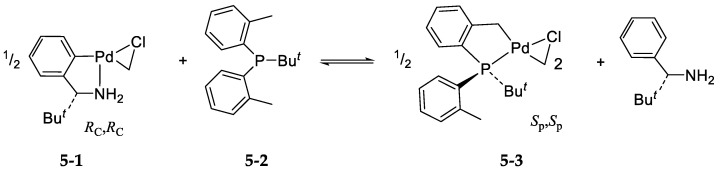
The exchange between chiral **5-1** with a primary amine and prochiral phosphine **5-2**.

The exchange reaction between chiral complex **5-1** with a primary amine and the prochiral phosphine **5-2** performed in a toluene/HOAc mixture at 60 °C for 1 h afforded the racemic *C,P*-dimer **5-3** in a 71% yield (Scheme 9). However, when the reaction was run in aprotic medium at room temperature, the yield dropped to 48% but the asymmetric induction was achieved, the enantiomeric excess (ee) of **5-3** being 72–91% depending on the reaction duration (96–140 h) [34].

**Figure 5:**
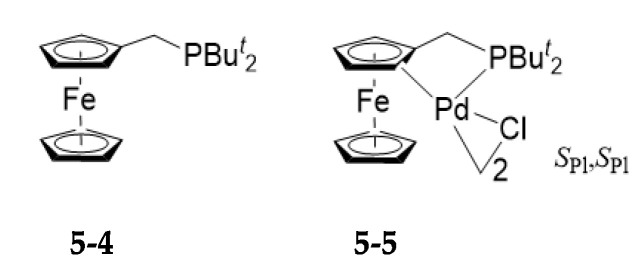
Prochiral **5-4** and its cyclopalladation product **5-5**.

Chiral primary amine complex **5-1** is stereochemically advantageous compared to other chiral palladacycles. Its α-methyl-substituted analogue, which is less bulky than **5-1**, reacted with a 66% yield of **5-3** but with just 13% ee. The complex incorporating *N*-methyl-α-Bu^t^ derivative, i.e., secondary amine, was practically unreactive (just 8% yield with less than 9% ee at 60 °C for 132 h) [34]. Attempts to transfer chirality from **5-1** at ferrocenylmethylphosphine **5-4**, i.e., to synthesize a planar chiral palladacycle (Figure 5), were much less successful under the same conditions as for **5-2**. Planar chiral *C,P*-dimer (*S*_Pl_,*S*_Pl_)-**5-5** was isolated with only a moderate enantiomeric excess of 44% at 26% chemical yield [34].

**Scheme 10:**
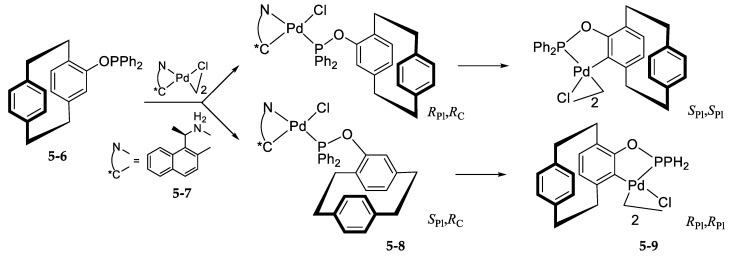
An approach to palladacycles with planar chirality based on the exchange of cyclopalladated ligands. Adapted from [35].

The asymmetric ligand exchange affording species with planar chirality was accomplished in the presence of acidic co-solvent. A mixture of two diastereomeric complexes **5-8** was generated in situ by reacting the racemic phosphinite **5-6** (derived from [2.2]paracyclophane) with cyclopalladated dimer (*R*,*R*)-**5-7**. The reaction mixture was separated to give both isomers, (*R*_Pl_,*R*_C_)-**5-8** and (*S*_Pl_,*R*_C_)-**5-8**, in diastereomerically pure form (>98% de), the isolated yields being 77% and 85%, respectively (Scheme 10). The final step of the exchange was performed in toluene in the presence of glacial acetic acid. Heating of each of the diastereomers, (*R*_Pl_,*R*_C_)-**5-8** and (*S*_Pl_,*R*_C_)-**5-8**, afforded two enantiomers of the target dimers **5-9**, (*S*_Pl_,*S*_Pl_) and (*R*_Pl_,*R*_Pl_), in ca. 50% yields [35].

## 6. The Mechanism of Palladium *N/N* Ligand Exchange

### 6.1. Thermodynamics of Palladium(II) Exchange

**Scheme 11:**
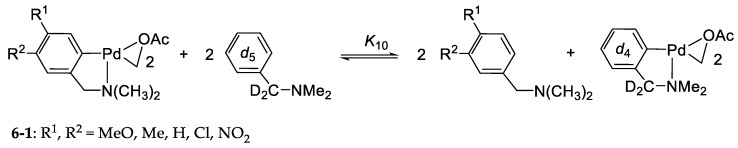
Investigation of thermodynamics of palladium(II) exchange.

A quick glance at exchange reactions in Table 1 suggests that palladium(II) has a tendency to migrate from electron-rich to electron-poor ligands. The trend might first seem controversial since Pd^II^ is, by and large, an electrophilic center with respect to aromatic and aliphatic C–H bonds [2]. This tendency was quantified by the example of reaction in Scheme 11 run in D_3_CCO_2_D-CDCl_3_ mixtures at 55 °C [36]. 

**Figure 6:**
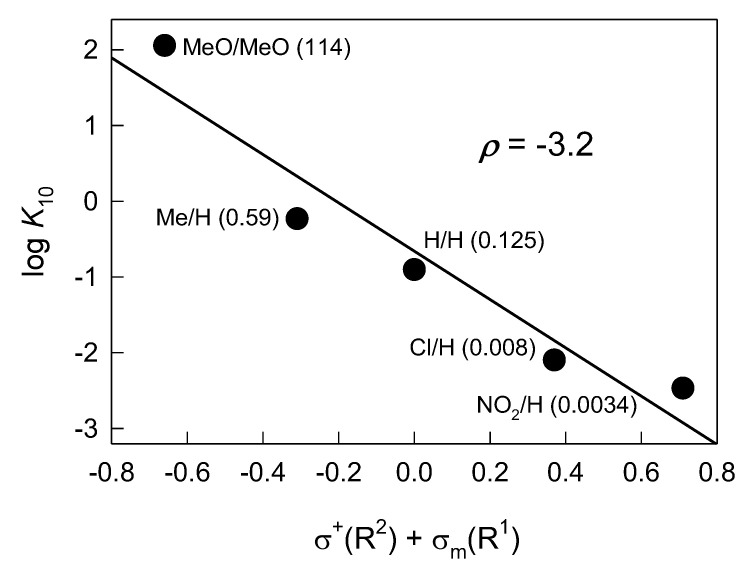
The Hammett plot for reaction in Scheme 11 created using composite σ values (see text for details). The equilibrium constants *K*_10_ (55 °C, in D_3_CCO_2_D-CDCl_3_) are shown in parenthesis after R^1^/R^2^ substituents in **6-1**. The data are from [38].

**Scheme 12:**
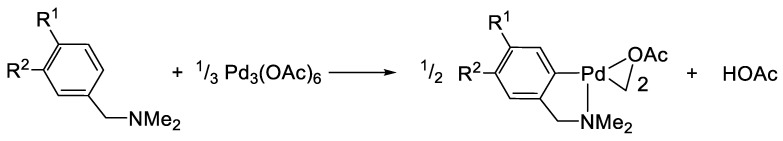
Cyclometalation of *N,N*-dimethylbenzylamines by palladium(II) acetate.

The values of equilibrium constants *K*_10_ (Figure 6) show convincingly that the equilibrium in Scheme 11 is shifted to the right for electron-rich ligands and to the left for electron-poor ligands. The slope of the Hammett plot in Figure 6-1, when log *K*_10_ were plotted against composite σ values (σ^+^ for R^2^ + σ_m_ for R^1^), was −3.2 identifying a formally counter-intuitive *nucleophilic* nature of Pd^II^ in the ligand exchange! Objectives for such a peculiar case were unveiled through detailed kinetic investigations of not just the ligand exchange reaction in Scheme 1 but of the cyclopalladation of ring-substituted *N,N*-dimethylbenzylamines by palladium(II) acetate, Scheme 12, performed both in acetic acid and aprotic chloroform [37]. 

### 6.2. Kinetics and Mechanism of Palladium(II) Exchange

**Figure 7:**
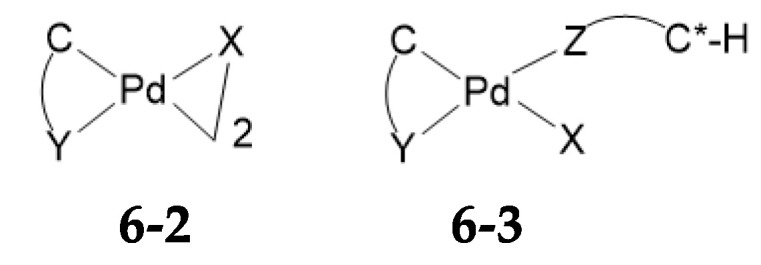
Structures of **6-2** and **6-3**.

Incoming ligands involved in the ligand exchange all contain a donor center that is capable of converting palladium(II) dimers into monomeric species, if the exchange occurs as shown in Scheme 5, Scheme 6, Scheme 7, Scheme 8 and Scheme 9 and Scheme 11. The monomerization by ligands such as pyridine may occur very quickly [39], and a starting palladium(II) complex to consider may be either **6-2** dimer or **6-3** monomer (Figure 7).

**Scheme 13:**
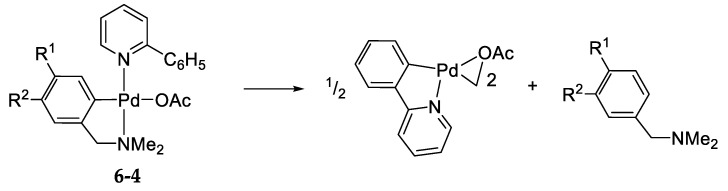
Intramolecular process in the case of 2-phenylpyridine.

Therefore, two corresponding systems were explored in detail, i.e., reactions of **3-2** with 2-phenylpyridine and azobenzene. Equilibrium studies performed in acetic acid in a broad temperature range indicated that the exchange with 2-phenylpyridine starts with **6-4** monomer (Scheme 13), though in the case of azobenzene, dimer **6-2** should be considered a starting material. 

**Figure 8:**
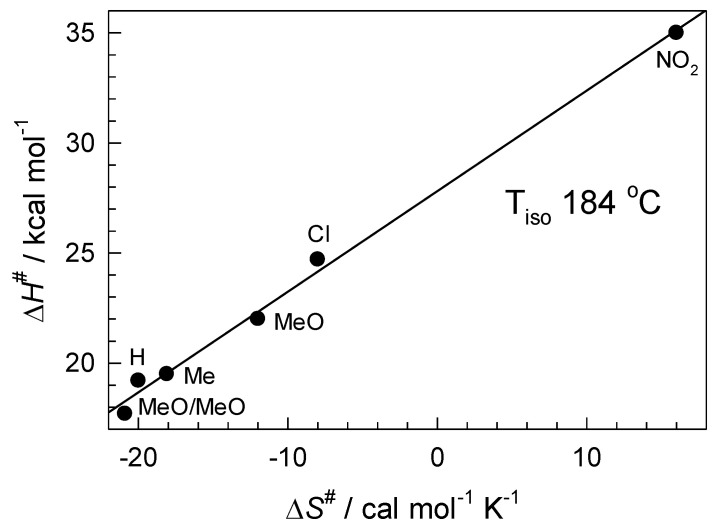
The isokinetic dependence for reaction in Scheme 13 obtained using variable starting complexes **6-4** (see text for details). R^1^ (R^2^ = H) substituents in the starting cyclopalladated complexes **6-4** accompany each data point; MeO/MeO stands for R^1^ = R^2^ = MeO. The data are from [38].

Despite such a variation, identical rate laws were established for the two cases. The exchange followed first-order kinetics in the palladium(II) material and zero-order kinetics in either 2-phenylpyridine or azobenzene. The zero-order was fully anticipated for 2-phenylpyridine, since the reaction of **6-4** is intramolecular (Scheme 13), though a similar observation for azobenzene pointed to a dissociative mechanism of the exchange with a rate-limiting departure of the cyclopalladated fragment of **6-2**. All kinetic data for process (12) are also consistent with a dissociative mechanism which holds in the entire series of Pd^II^ complexes **6-4** as suggested by a perfect isokinetic dependence shown in Figure 8. The regression coefficient equals 0.99 and the calculated isokinetic temperature of 184 °C is far away from a temperature range used for collecting experimental data [38]. 

Very high values of the enthalpy of activation Δ*H*^≠^ (25–35 kcal mol^−1^), particularly for **6-2** with electron-withdrawing substituents R^1/2^, served strong evidence for a dissociative mechanism of the exchange which was also consistent with the value of a solvent kinetic isotope effect *k*_H_/*k*_D_ = 1.80 measured in H_3_CCO_2_H and D_3_CCO_2_D for the reaction in Scheme 13. It was 1.08 in the case of azobenzene. The reactivity gap of three-orders of magnitude for complexes **6-2**, which provided the Hammett ρ value of −2.9 against σ_m_, is also worth mentioning. The absolute value of ρ is high for palladium complexes and this fact is consistent with a bond breaking in the transition state. 

**Scheme 14:**
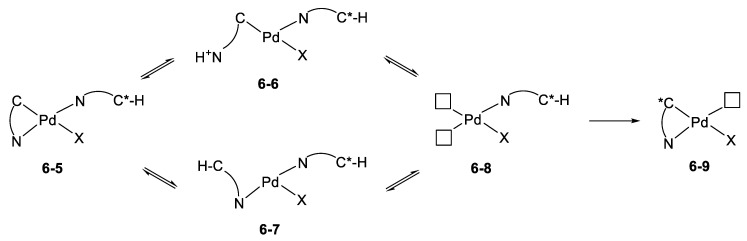
Intimate features of the dissociative mechanism of the ligand exchange involving two *N*-donor ligands. Adapted from [38].

The dissociative mechanism of reaction in Scheme 13 implies that the initially cyclopalladated ligand departs first and this step is rate limiting. The departure of a bidentate ligand creates a co-ordinatively unsaturated or solvento electrophilic palladium(II) intermediate **6-8**, which cleaves a proper C–H bond of the incoming ligand (Scheme 14). The intermediate may attack the C–H bond of the dissociated ligand as well and it is a question of a thermodynamic control where Pd^II^ finds itself after completion of the exchange (see below). Intimate mechanistic options for the departure are associated with the question of which bond of the palladacycle is cleaved first, i.e.*,* N–Pd or C–Pd (Scheme 14). The C–Pd variant agrees with the principle of microscopic reversibility because cyclopalladation starts with an *N*-co-ordination of incoming ligand followed by C–H bond palladation [2,37]. 

**Scheme 15:**
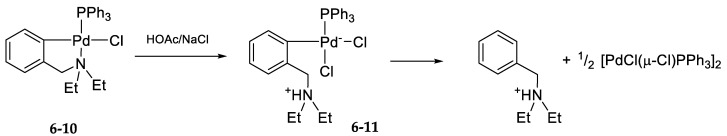
Evidence in favor of the intermediacy of **6-6**.

Evidence in favor of the intermediacy of **6-6** was brought about by isolation of the zwitter-ionic complex **6-11** which is similar to postulated intermediate species **6-6**. It is formed from **6-10** in acetic acid in the presence of NaCl [40]. Zwitter-ion **6-11** is unstable and collapses into final products after the protonolysis of the C–Pd bond (Scheme 15).

It is hard to say which of the two pathways in Scheme 14 is more likely. The values of *k*_H_/*k*_D_ are consistent with both pathways, though higher values better support the intermediacy of **6-7** because the acidic proton is likely more involved in the C–Pd bond cleavage rather than in the cleavage of the co-ordinative N–Pd bond. It does not seem improbable that a contribution of either pathway varies as electronic properties of the leaving ligand change.

**Scheme 16:**
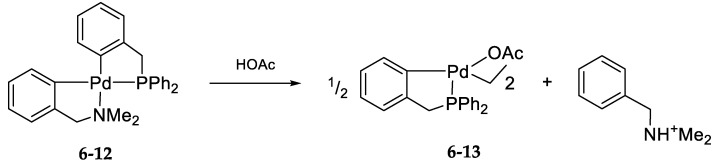
Evidence against the “associative” mechanism of the ligand exchange.

The “associative” mechanism of the ligand exchange with the intermediacy of a bis-cyclopalladated complex such as **6-12** was ruled out due to a high protic instability of such species. Traces of acetic acid destroy complex **6-12** rapidly to produce an anticipated mixture of acetato-bridged phosphine complex **6-13** and protonated *N,N*-dimethylbenzylamine (Scheme 16) [41].

### 6.3. “Mechanism” of Thermodynamic Control

**Scheme 17:**
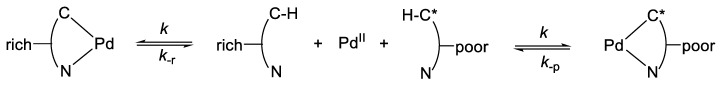
Kinetic rationalization of the thermodynamic preference of Pd^II^ for electron-poorer ligands in the exchange of cyclopalladated ligands (see text for details).

Reasons for the thermodynamic preference of Pd^II^ for electron-deficient units in the ligand exchange were disclosed through kinetic investigations of two processes, viz. (i) the exchange itself and (ii) direct cyclopalladation of ring-substituted *N,N*-dimethylbenzylamines by Pd^II^ acetate, Scheme 12 in chloroform and acetic acid as solvents [37]. In chloroform, the cyclopalladation is preceded by a rapid conversion of the cyclic trimer Pd_3_(OAc)_6_ to the reactive monomer [Pd(OAc)_2_(N~CH)_2_], where N~CH is incoming *N,N*-dimethylbenzylamine. The rate-limiting C–H bond cleavage occurs after reversible dissociation of N~CH to form [Pd(OAc)_2_(N~CH)] as a reactive intermediate. The intramolecular C–H cleavage is a typical base-assisted electrophilic aromatic substitution in CHCl_3_ suggested by the Hammett ρ value of −1.6, the kinetic isotope effect *k*_H_/*k*_D_ of 2.2 [37]. This mechanistic conclusion was confirmed experimentally [42,43,44,45,46] and theoretically [47,48,49,50]. When reaction in Scheme 12 is run in pure acetic acid, the cyclopalladation is reversible, and the second-order rate constants are practically independent of the nature of ring substituent in the *N,N*-dimethylbenzylamine ring. Palladium(II) does not behave as a true electrophile because there is no kinetic preference of Pd^II^ for any benzylamine ligand in this solvent. The experimental facts briefly summarized in this paragraph plus the strongest sensitivity of the reaction to the nature of R^1^ and R^2^ provide a straightforward explanation of the thermodynamic preference of Pd^II^ for electron-poorer ligands (Scheme 17).

The reversible cyclopalladation occurs with similar rate constants *k* for electron-rich and electron-poor incoming ligands in acetic acid. The rate constants for the dissociation of cyclopalladated ligands, in contrast, are extremely sensitive to the electronic effects and *k*_-r_ >> *k*_-p_. Therefore, the corresponding equilibrium constants *K* = *k*/*k*_-r/p_ should be larger for electron-poor incoming ligands! Thus, the selectivity is controlled by the rates of acid-induced dissociation of cyclopalladated ligands. 

**Scheme 18:**
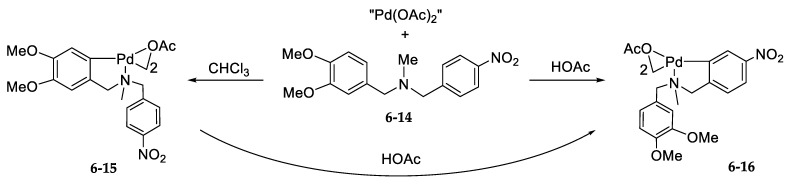
Synthetic verification of mechanistic conclusions on cyclopalladation and ligand exchange involving acetate palladium(II) complexes of *N,N*-dimethylbenzylamine derivatives.

The conclusions derived from kinetic and thermodynamic data were verified by studying reactivity trends of “bifunctional” benzylamine **6-14** in reactions with palladium(II) acetate carried out in two media, viz. aprotic chloroform media and acetic acid [38]. The chemistry is shown in Scheme 18. Treatment of **6-14** with Pd^II^ in CHCl_3_ results in the predicted activation of the C–H bond of the electron-rich “MeO” ring to afford the acetato-bridged complex **6-15**. When the reaction is carried out in acetic acid, complex **6-16,** a positional isomer of **6-15,** is obtained in which Pd^II^ is bound to the nitro-substituted ring. The formation of **6-15** and **6-16** also suggests that a product distribution is governed by the electronic density at aromatic nuclei. 

**Scheme 19:**
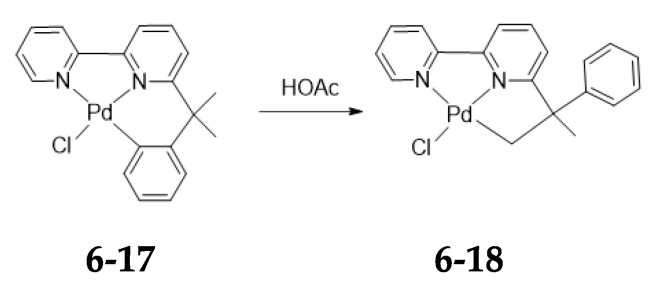
Intramolecular conversion of **6-17** to **6-18**.

When **6-15** dissolved in acetic acid is heated, a thermodynamic preference for electron-deficient aromatic units finds extra evidence as **6-15** rearranges into **6-16**. The **6-15** to **6-16** rearrangement exemplifies an intramolecular version of the ligand exchange, which was also described for cyclopalladated thioacetamides [51] and 6-(2-phenylpropan-2-yl)-2,2′-bipyridine [52]. In the latter case, compound **6-17** with Pd^II^ bound to the *sp*^2^ carbon transforms into **6-18** having the C(*sp*^3^)–Pd bond (Scheme 19). 

### 6.4. A Mechanistic Comment Regarding Section 5

The mechanistic information obtained in acidic media using tertiary amine complexes **3-2** as a palladium(II) source and *N*-donor targets as incoming ligands provides solid grounds for the dissociative ligand exchange pathway. If it were the case for all cyclopalladated complexes and all incoming ligands, the asymmetric version of exchange of cyclopalladated ligands described in Section 5 would not be possible. The asymmetric induction is so far observed when (i) chiral pallada(II)cycle incorporating a primary amine is used and (ii) palladium(II) cleaves C–H bonds of *P*-donor ligands. The tertiary vs. primary dilemma in cyclopalladation of benzylamines was known since 1968 [53]. Primary benzylamines refused to be cyclopalladated for quite a long time until proper reaction conditions were disclosed [54,55,56] followed by a mechanistic study [57]. The tertiary vs. primary paradox was accounted by a stronger binding of primary amines to transition metal complexes causing troubles in generating co-ordinatively unsaturated or solvento intermediates such as [Pd(OAc)_2_(N~CH)] [2]. Stronger binding of primary amines allows them to stay co-ordinated to palladium intermediates after C–Pd bond cleavage and thus to serve as asymmetric inductors during C–H bond activation of incoming *P*-donor ligands. The former seem to play an important role as well. Being softer ligands, they presumably make palladium(II) a softer acid and this could favor moving hydrogen as a hydride rather than as a proton. Correspondingly, the intermediacy of Pd^IV^ as a result of oxidative addition should be assumed. Evidence for electrophilic to oxidative addition mechanistic changeover in Pd^II^ chemistry was presented [58] and explored in detail by Canty et al. [59,60].

## 7. Processes Mechanistically Relevant to Palladium Ligand Exchange 

### Palladation by η^3^-Allyl Palladium(II) Dimers

Allylic complexes [PdCl(η^3^-allyl)]_2_ are resistant to protolytic cleavage [58]. The departure of the η^3^-bonded allyl in acetic acid takes place in the monomeric phosphine complex [PdCl(η^3^-2-PhC_3_H_4_)(PPh_3_)] when the latter is thermolyzed at 80 °C in the presence of LiCl (Equation (1)).

[PdCl(η^3^-2-PhC_3_H_4_)(PPh_3_)] + HOAc + LiCl → ½ [PdCl(μ-Cl)(PPh_3_)]_2_ + H_2_C=CMePh + LiOAc (1)

**Scheme 20:**
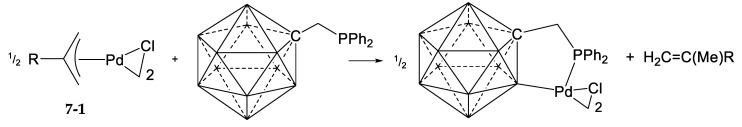
The migration of Pd^II^ from η^3^-allylic complex **7-1** to *B,P*-carboranyl ligand.

When **4-2a** was used instead of PPh_3_, the migration of Pd^II^ from η^3^-allylic complex **7-1** to *B,P*-carboranyl ligand occurred (Scheme 20) [26].

The co-ordinated tertiary phosphines obviously increase the lability of η^3^-allyls since no palladium migration is observed in the case of 2-phenylpyridine, although the latter readily splits chloro-bridges of [PdCl(η^3^-2-PhC_3_H_4_)]_2_. The reaction course is independent of whether the η^3^-allyl dimer and **4-2a** or the monomer [PdCl(η^3^-allyl)(**4-2a**)] are used as starting materials though the ratio of B3 and B4 isomers formed differed from that observed in reaction in Scheme 7 in acetic acid solvent. Interestingly, no migration of palladium(II) from η^3^-allyls to azobenzene, 2-phenylpyridine, *N,N*-dialkylbenzylamines as well as the exchange between dimeric η^3^-allyls and corresponding allylic alkenes was observed [26].

## 8. Exchange in the Platinum Series 

### 8.1. η^3^-Allyls as Leaving Ligands

**Scheme 21:**
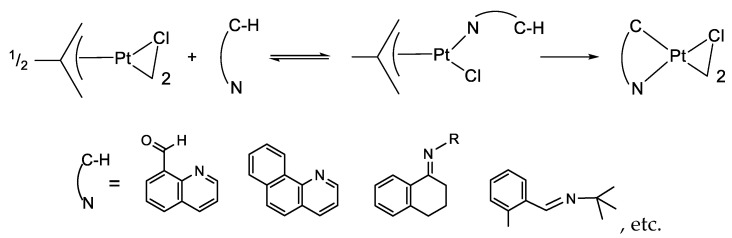
Platinum(II) transfer from allylic to *N*-donor ligands.

Though palladium(II) does not migrate from allyls to *N*-donor ligands as it does in reaction in Scheme 20, platinum(II) allylic chloro-bridged dimers react differently and migrate readily to form *N*-donor platinacycles (Scheme 21) [61,62,63,64,65,66,67] or *P*-donor pincer Pt^II^ complexes [68]. These exchange reactions occur in aprotic solvent and acids are not needed for triggering the platinum(II) migration, which is an indication of a different mechanism. Much like the palladium case, the first step is the chloro-bridge cleavage to form the corresponding monomeric species. It is followed by the transformation of the latter into the reaction products probably involving η^3^ → η^1^ rearrangement of the allylic ligand [61] needed for the creation of a vacant co-ordination site at a platinum center.

**Scheme 22:**
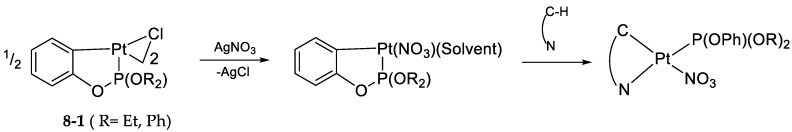
Silver(I) nitrate induced the exchange of cycloplatinated ligands involving the migration from *P*-phenylphosphite to *N*-donor ligands.

The next example implies a mechanistic variety in the exchange in platinum(II) versus palladium(II) complexes. Scheme 22 shows the Pt^II^ migration from cycloplatinated phenylphosphites **8-1** to *N*-donor molecules listed in Scheme 21. The reactions proceed in the presence of AgNO_3_ in a CH_2_Cl_2_/MeOH mixture. Silver(I) abstracts chloro ligands and activates Pt^II^ with respect to C–H bonds of incoming *N*-donor ligands. 

### 8.2. True Exchange of Cycloplatinated Ligands

The co-ordination/organometallic chemistry of Pd^II^ and Pt^II^ has much in common, though platinum(II) reactions are run by ca. 4 orders of magnitude slower [69,70]. Cyclometalated ligands as strong σ-donors are known to elevate the reactivity of Pt^II^ complexes to the level of their Pd^II^ counterparts [39,71]. Correspondingly, the palladium exchange usually occurs via *C,N*-ligand-free intermediates, whereas the platinum case takes place with the intermediacy of the Pt(*C,N*-ligand) solvento species [72].

**Scheme 23:**
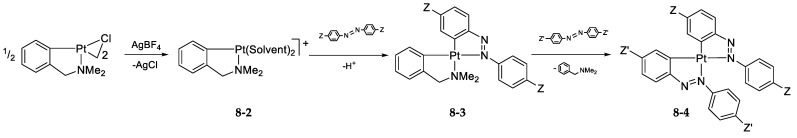
Conversion of **8-3** to **8-4** as a true example of the exchange of cycloplatinated ligands. Adapted from [72].

An illustrative example is presented in Scheme 23 [72]. Chloro ligands of the platinum analogue of **3-1** were first removed by AgBF_4_ dissolved in acetone (*cf.* with Scheme 21) and solvento complex **8-2** produced was reacted with excess azobenzene in CH_2_Cl_2_ or benzene. The heteroleptic bis cycloplatinated complex **8-3** is formed first followed by its gradual transformation into azobenzene species **8-4** with either identical or different cyloplatinated ligands. The **8-3** → **8-4** rearrangement exemplifies the exchange of cyclometalated ligands in the platinum series. 

The **8-3** → **8-4** conversion is initiated by traces of acids. Though the reaction was run in acid-free organic solvent, acid is produced during the **8-2** → **8-3** transformation as the cycloplatination of azobenzene occurs as an electrophilic substitution with a liberation of a proton. When **8-3** was isolated and then treated with azobenzenes in the presence of stoichiometric amounts of triflic acid, the **8-3** → **8-4** conversion readily occurred, providing a synthetic route to heteroleptic bis cyclometalated platinum(II) products. 

**Scheme 24:**
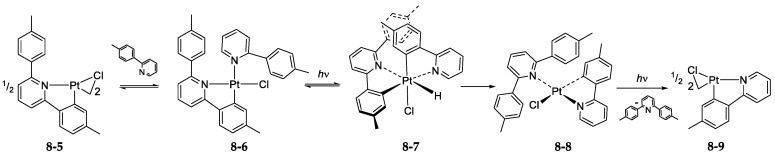
Photochemical **8-5** → **8-9** transformation.

A photochemical version of the platinum exchange was also described [73]. It starts with **8-5** in acetone in excess of 2-(4′-tolyl)pyridine. Irradiation by visible light of the monomeric Pt^II^ complex **8-6** induces the C–H bond cleavage via oxidative addition to afford Pt^IV^ derivative **8-7**, which first collapses to **8-8** followed by its photochemical conversion to **8-9**. The overall photochemical **8-5** → **8-9** transformation in Scheme 24 is identical to the exchange of cyclopalladated ligands listed in Table 1. A carbene complex like **8-7** was also reported for a 1,2,3-triazolylidene derivative [74]. 

**Scheme 25:**
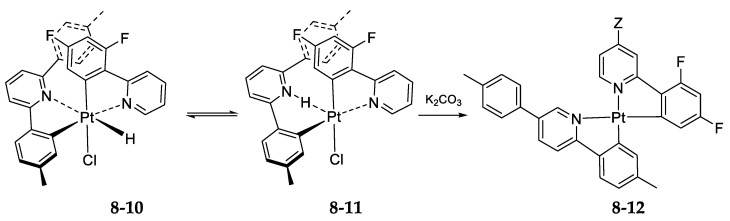
Approach to heteroleptic doubly cycloplatinated complex **8-12**.

Interestingly, if 2-(2′,4′-difluorophenyl)pyridine is used instead of 2-(4′-tolyl)pyridine, it is possible to divert the Pt^IV^ intermediate **8-10** to **8-11** first, which transforms to heteroleptic doubly cycloplatinated complex **8-12** (Scheme 25) [73]. There is a striking similarity of **8-12** with complexes **8-4**. The authors ignored to mention this fact. 

### 8.3. Intramolecular Exchange of Cycloplatinated Ligands

**Scheme 26:**
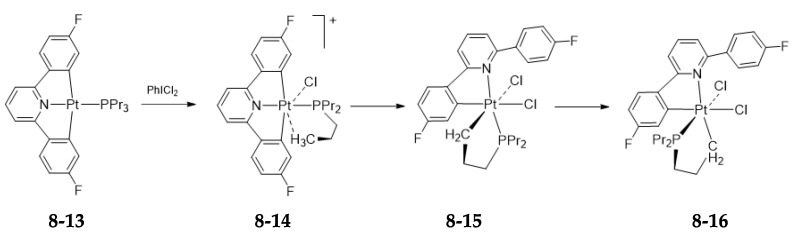
Transformation of **8-13** to **8-16**.

The oxidation of the doubly cycloplatinated 2,6-di(4′-fluorophenyl)pyridine derivative **8-13** with the electrophilic oxidant iodobenzenedichloride yields five-co-ordinate species **8-14** with an agostic interaction that subsequently undergoes the exchange of cycloplatinated ligands to give **8-15** with a singly cyclometalated pyridine and a cyclometalated phosphine which isomerizes into **8-16** (Scheme 26) [75]. Similar transformations were reported for P(*^n^*Bu)_3_ [76] and tri(*o*-tolyl)phosphine [77] analogues of **8-13**. 

**Figure 9:**
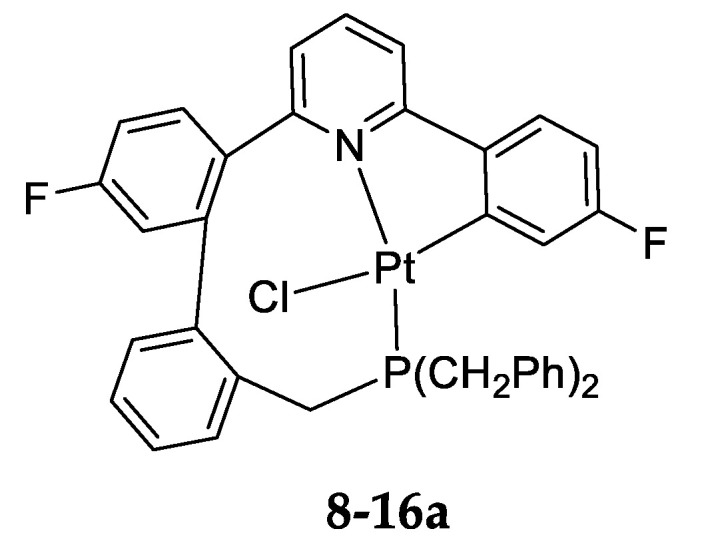
Structure of **8-16a**.

More complex chemistry was revealed using the tribenzylphosphine ligand [78]. The oxidation of the tribenzylphosphine analogue of **8-13** with PhICl_2_ led, as a first step, to a triply cyclometalated Pt^IV^ species, which underwent intramolecular reductive elimination to afford a Pt^II^ complex **8-16a** (Figure 9) with a nine-membered platinum-containing ring.

### 8.4. Pincer-Pincer Ligand Exchange in Cycloplatinated Complexes

**Scheme 27:**
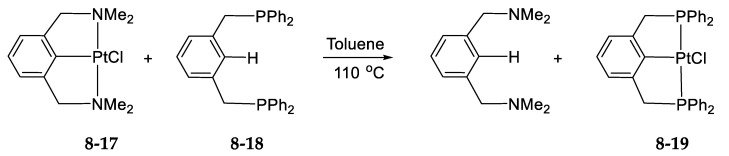
The ligand exchange in Pt^II^ pincer complexes.

No acid initiation is needed for launching the ligand exchange in Pt^II^ pincer complexes (Scheme 27) which occurs cleanly in toluene at 110 °C [79]. No exchange was detected when **8-17** reacted with *N*-donor analogues of **8-18** [80]. Product **8-19** was isolated in 98% yield after refluxing the reaction mixture for 3 days. 

**Scheme 28:**
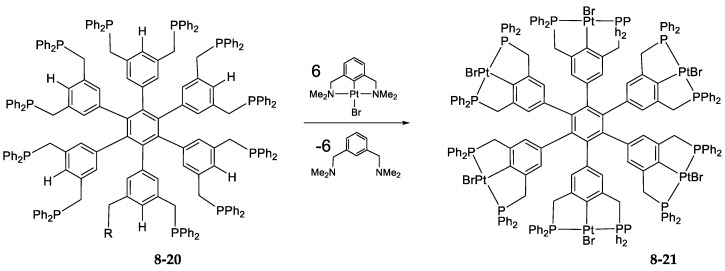
Synthesis of hexacycloplatinated dendrimer-like compound **8-21**.

Such extraordinary features of the reaction in Scheme 27 were further applied to a synthesis of a more sophisticated hexacycloplatinated dendrimer-like compound **8-21** (Scheme 28) using the bromo variant of **8-17** (30% yield after refluxing for 7 days) [81,82].

**Scheme 29:**
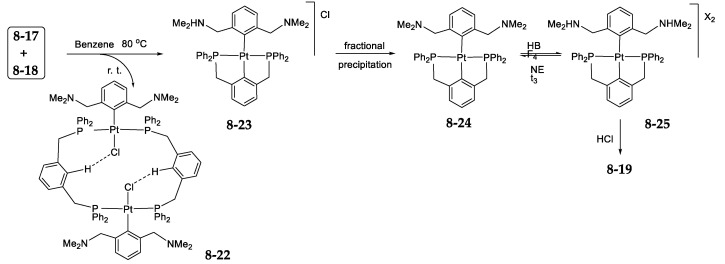
Intermediates *isolated* and *characterized* prior to the formation of the final product **8-19**.

The reaction in Scheme 27 is not fast. It occurs without acidic additives under reflux and therefore is a setup for trapping intermediates formed and establishing its mechanism [79]. When the reaction of **8-17** with **8-18** was carried out in benzene at room temperature, a white powder formed was identified and structurally characterized as dimetallic complex **8-22** (Scheme 29). At 80 °C, complex **8-23** was isolated, which after fractional precipitation and addition of pentane to the solution in CH_2_Cl_2_ transformed into a mixture of **8-24** and **8-25**. The quantitative formation of **8-25** occurs on addition of HBF_4_ but a large excess of acid, e.g., HCl, causes Pt–C bond cleavage affording **8-19**.

**Scheme 30:**
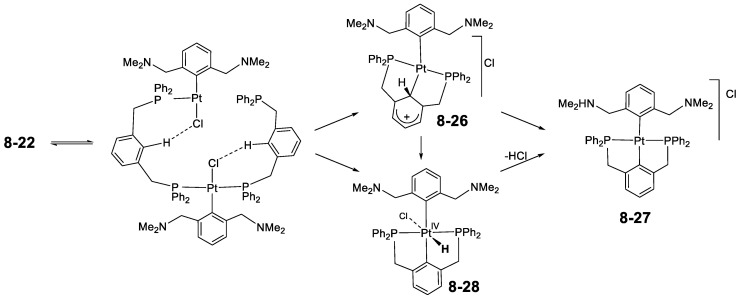
Mechanistic details and *postulated* intermediates for the conversion of **8-22** to **8-27**.

The mechanistic details proposed are outlined in Scheme 30. Assuming that **8-22** is on the reaction co-ordinate, a reversible cleavage of a Pt–P bond affords the reactive intermediate, which is viewed as an electron-deficient co-ordinatively unsaturated 14e metal center. The latter is involved in an electrophilic aromatic substitution affording an **8-26** arenium intermediate, but proton abstraction by basic nitrogen of a dimethylaminomethyl arm affords **8-27**. 

The authors also considered a mechanistic option with an intermediacy of a platinum(IV) octahedral hydride complex **8-28** formed from the 14e intermediate via the oxidative addition of aryl C–H bond to Pt^II^ or from **8-26** as a result of 1,2-sigmatropic H shift (Scheme 30) [79]. Though it is difficult to imagine how the reductive elimination of HCl could occur from **8-28** where chloride and hydride are positioned trans to each other, the reductive elimination of 1,3-(Me_2_NCH_2_)_2_C_6_H_4_ affording the final product of the reaction, complex **8-19**, appears to be a suitable pathway. Otherwise, one needs to postulate that the dissociation of the diamine from **8-27** takes place due to the intramolecular proton delivery from its protonated Me_2_NH^+^CH_2_ arm. 

### 8.5. Exchange of Cyclometalated Ligands or Transcyclometalation?

When one ligand substitutes another ligand in the co-ordination sphere of a metal complex, this is a ligand substitution. Naturally, when one cyclometalated ligand is replaced by another cyclometalated ligand, this is a substitution of cyclometalated ligand. Therefore, there were no doubts to name the reaction in Scheme 1 as the exchange of cyclometalated ligands in early eighties [8], and it was delightful to see that the ligand exchange strategy was extended at other systems by other scientists. However, there was a slight motif of disappointment when the initially suggested term for reaction in Scheme 1 was neglected and replaced by transcyclometalation in analogy to transesterification reactions [79,83]. Closing eyes on the fact that renaming obscures a contribution of inventors, this particular term is chemically incorrect.

*Transmetalation* in organometallic chemistry is a reaction involving two metal centers, Equation (2) (https://en.wikipedia.org/wiki/Transmetalation#:~:text=Transmetalation%20(alt.,%2C%20or%20pseudo%2Dhalogen%20group). One metal complex reacts with another, the overall result being the redistribution of ligands around two metal centers involved [84]:

M_1_–R + M_2_–R′ → M_1_–R′ + M_2_–R (2)

**Scheme 31:**
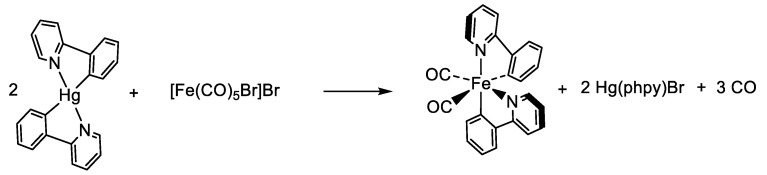
Synthesis of ferrocycles starting from symmetric organomercury precursors.

Here, R and R′ can be, but are not limited to, an alkyl, aryl, alkynyl, allyl, halogen or pseudo-halogen group. The term transcyclometalation should therefore be applied a different transformation between a cyclometalated complex and a complex at which the cyclometalated moiety is being transferred. Transcyclometalation in Scheme 31 is an illustrative example used for the synthesis of ferrocycles starting from the symmetric organomercury precursors [85]. Transmetalation or transcyclometalation was developed for ruthenium and osmium complexes [86,87,88,89] and then adapted for the synthesis of more difficult to make iron(II) species (Scheme 31).

**Scheme 32:**
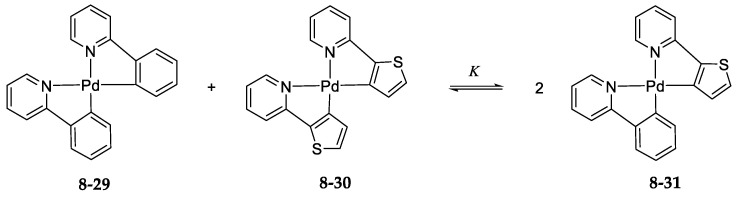
Example of true transcyclometalation.

It is worth noting that the true transcyclometalation was actually reported by von Zelewsky et al. for homoleptic bis-cyclomatelated Pd^II^ complexes (Scheme 32) [90]. A mixture of the two homoleptic species **8-29** and **8-30** affords in chloroform solution over an extended period of time the heteroleptic species **8-31** and vice versa. Equilibrium can be reached from both sides in a matter of ca. 50 h at 20 °C. The equilibrium constant *K* is approximately 1.8 and is lower than the statistical equilibrium constant, indicating that the homoleptic complexes are thermodynamically slightly preferred [90]. The exchange is independent of solvent purity (CDCl_3_), since highly purified chloroform gave the same results, but it is considerably slowed down in CH_2_Cl_2_. The exchange takes place between **8-29** and the benzo[*h*]quinoline analogue of **8-30**, but with a considerably slower rate.

**Scheme 33:**
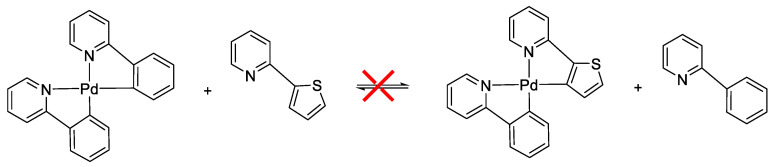
Evidence against “free ligand” pathway.

Some mechanistic insight was obtained by examining the possibility of reaction in Scheme 32. Mixed complexes were not formed in this way (Scheme 33), excluding the possibility of a “free ligand” pathway for the exchange reaction [90].

**Scheme 34:**
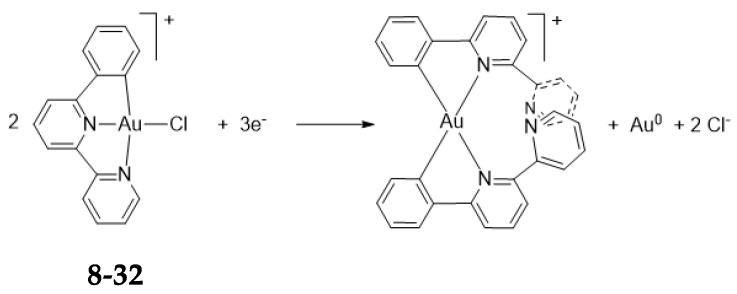
The electrochemical reduction of **8-32**.

Bimolecular reaction in Scheme 34 involving two identical gold(III) cyclometalated complexes [91] can also be classified as transcyclometalation though it is a reductive process. The electrochemical reduction of **8-32** promotes the redistribution of the cycloaurated ligand between two gold centers. The stoichiometry of reaction in Scheme 34 is much more consistent with Equation (2) than with that in Scheme 27.

**Scheme 35:**
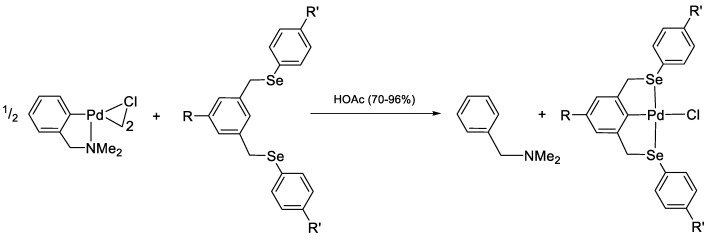
Synthesis of selenium pincer complex.

Similarities between the reaction in Scheme 35 [92] and that in entry 13 of Table 1 in terms of a general strategy, palladium(II) source and reaction conditions are striking. Nevertheless, the authors preferred to use the term “transcyclometalation” and, regrettably, did not acknowledge original publications where similar processes were introduced. 

The original title of this account was planned to be “Exchange of Cyclometalated Ligands or Transcyclometalation”. The Reviewer recommended to drop the term transcyclometalation assuming that new readers should use the correct terminology from the beginning. Needless to say, such a modification was very enthusiastically made!

## 9. The Ligand Exchange with Pincer Ruthenium Complexes

**Scheme 36:**
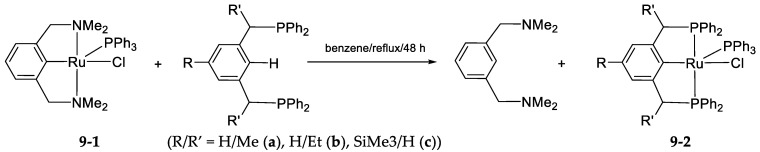
Ruthenium(II) migration from nitrogen to phosphorus pincer ligand.

Ruthenium(II) migration from a nitrogen to phosphorus pincer ligand occurs in refluxing benzene without other additives as in Scheme 36 [93]. NMR data suggest that the ligand exchange occurs nearly quantitatively. Isolation of complexes **9-2a,b** was achieved by precipitation with cold hexane from a reaction solution in CH_2_Cl_2_ in 70 and 40% yield, respectively [93]. The reaction is particularly advantageous for the synthesis of **9-2c** because direct cyclometallation of the free phosphorous ligand **9-1c** by [RuCl_2_(PPh_3_)_3_] was accompanied by the C–Si bond cleavage presumably by liberated HCl to produce **9-2a**. Partially fluorinated phosphine ligand 1,3-((C_6_F_5_)_2_PCH_2_)_2_C_6_H_4_ affords the product analogues to **9-2** in an 83% yield [94]. Pro-pincer ligand 1,3-(*^i^*Pr_2_PCH_2_)_2_C_6_H_4_ reacts similarly [95,96].

The potential of **9-1** in the ligand exchange to create multiple metal–carbon bonds was further illustrated by its reaction with **8-20** in refluxing benzene. The air-sensitive dark green hexaruthenium analog of **8-21**, which is structurally similar to **9-2**, produced first. The stability of the primary product was markedly enhanced by reacting it with the terpy ligand. The reaction was accompanied by a color change from dark green to dark red; it changed geometry around the ruthenium centers from distorted square-pyramidal (as in **9-2**) to octahedral. The complex became stable towards air and water [82]. The initially formed, untreated with terpy complex was tested as a homogeneous catalyst in the hydrogen transfer reactions of cyclohexanone, acetophenone and benzophenone to alcohols. Its activity per ruthenium center was of the same order of magnitude as that of **9-2** (R=R′=H) [81]. 

**Scheme 37:**
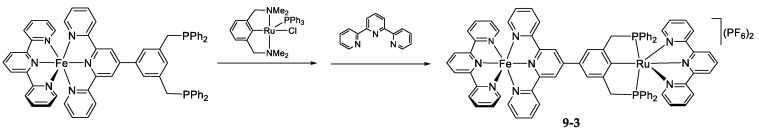
Preparation of iron-ruthenium bimetallic compounds.

The exchange of pincer cycloruthenated ligands is convenient for the preparation of iron-ruthenium bimetallic compounds such as **9-3** using **9-1** as a ruthenium(II) source (Scheme 37) [97]. The ligand exchange step was run in refluxing Thf/MeCN (8:3 *v*/*v*). 

**Scheme 38:**
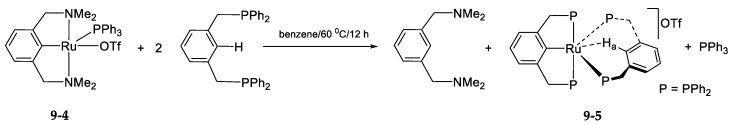
The *N*/*P* ligand exchange involving triflate ruthenium(II) pincer complexes.

**Scheme 39:**
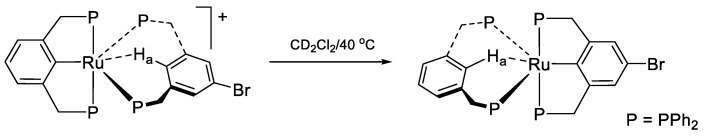
Migration of agostic hydrogen.

The course of the *N*/*P* ligand exchange involving ruthenium(II) pincer complexes changes with the replacement of chloro by triflate ligand in **9-1** [98]. Complex **9-4** reacts with two equivalents of the incoming phosphorous ligand **8-18** to form **9-5**, Scheme 38. Complex **9-5** was mechanistically challenging as it contained the agostic H_a_ atom which was shown to migrate from the neutral to the cyclometalated carbon as in Scheme 39.

An electrophilic aromatic substitution mechanism involving arenium-type intermediates, which are similar to **8-26**, were postulated [98]. If hydrogen travels as proton from one aromatic ring to another, the electrophilic mechanism is very likely because the proton migrates from the aromatic ring with an electron-releasing substituent to the unsubstituted aromatic ring (Scheme 39).

## 10. Other Examples of Exchange of Cyclometalated Ligands

### 10.1. Manganese(I)

**Scheme 40:**
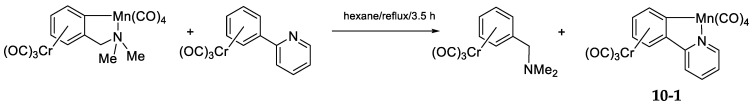
Exchange in cyclometalated manganese(I) carbonyl complexes.

The magnificent Djukic/Pfeffer duo explored the intermolecular exchange in cyclometalated manganese(I) carbonyl complexes containing electron-withdrawing η^6^-bound chromiumtricarbonyl unit [99]. The exchange given by Scheme 40 takes place cleanly under relatively mild conditions without acidic additives, and the cyclomanganated derivative of 2-phenylpyridine **10-1** was isolated in a 62% yield.

**Scheme 41:**
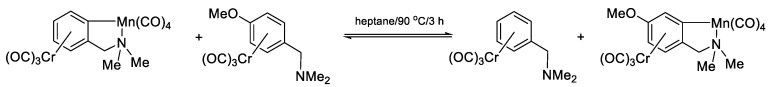
Migration of manganese(I) to an electron-rich ligand in cyclometalated carbonyl complexes.

The exchange in cyclometalated manganese(I) carbonyl complexes (Scheme 41), suggesting an electrophilic nature of this manganese exchange. The mechanistic options discussed included an oxidative addition to form a hydrido-Mn^III^ complex, a multicentered process and a radical-mediated pathway [100]. Similar migrations of the Mn(CO)_4_ were also reported [101,102].

### 10.2. Rhodium(III)

**Scheme 42:**
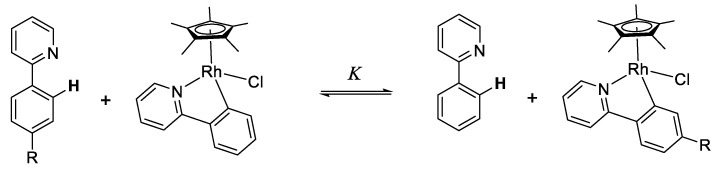
Intermolecular exchange of cyclometalated ligands in rhodium(III) complexes.

VanderWeide et al. proved that the intermolecular exchange of cyclometalated ligands exists among rhodium(III) complexes (Scheme 42) [103]. The reaction occurs in a methanol solvent buffered by 0.05 M NaOAc/HOAc, i.e., an acidic solvent component is essential. Just this fact underscores similarities between palladium(II) (Scheme 11) and rhodium(III) (Scheme 42). There are other common features as well. The Hammett ρ value for equilibrium constants *K* for equilibrium in Scheme 42 of +1.34 indicates that rhodium(III) has a preference for aromatic rings with electron withdrawing substituents. Palladium(II) behaves in a similar way.

A correlation was established between *K* and the *heterolytic* C–H bond strengths. Substrates with larger heterolytic C–H bond strengths (i.e., less acidic) are less favorable to activate than substrates with weaker heterolytic C–H bond strengths. Since substrates with electron-withdrawing groups have weaker heterolytic bond strengths, cyclometalation of these substrates by Rh^III^ is preferred [103]. 

### 10.3. Iridium(III)

**Scheme 43:**
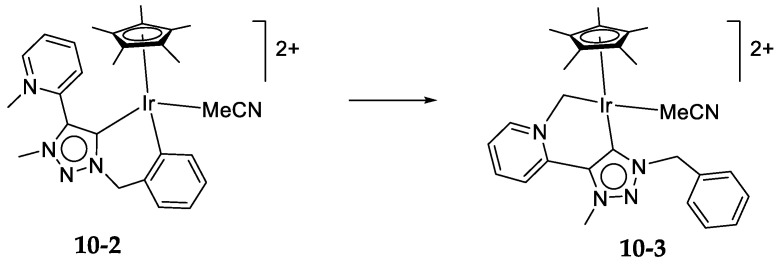
Intramolecular exchange of cyclometalated ligands in pyridinium triazolylidene iridium complexes.

The intramolecular exchange of cyclometalated ligands was observed in pyridinium triazolylidene iridium complexes (Scheme 43) [104]. Upon heating in MeCN solution, the cyclometalated intermediate **10-2** transformed exclusively to the C(*sp*^3^)–H bond activated complex **10-3**. The reaction in methanol or CH_2_Cl_2_ solvent is substantially faster than in MeCN. Rate measurements performed in CD_3_OD revealed a first-order kinetics in **10-2** and the *k*_H_/*k*_D_ kinetic isotope effect of 9.3 at 328 K (using **10-2** with *N*-bound CD_3_) supporting a C–H/D bond cleavage in the rate-determining step. The corresponding enthalpy of activation Δ*H*^≠^ is very large (33 kcal mol^−1^) and this makes the entropy of activation Δ*S*^≠^ large as well (23 cal mol^−1^ K^−1^). Such values of both *k*_H_/*k*_D_ and Δ*H*^≠^ are typically consistent with a concerted multicentered mechanism (σ-M–C bond metathesis) in which a leaving proton moves directly at a carbon of initial M–C bond [2].

**Scheme 44:**
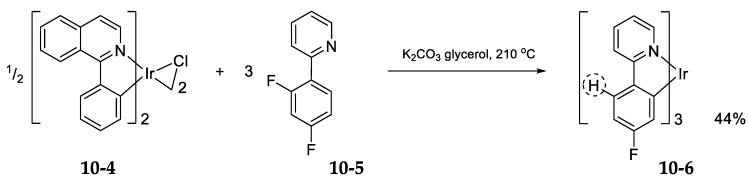
Exchange at iridium(III) chloro-bridged dimer **10-4**.

A mechanistic puzzle was brought about by the exchange at iridium(III) chloro-bridged dimer **10-4** produced from 1-phenylisoquinoline (Scheme 44) [105]. Complex **10-4** reacts with 2-(2,4-difluorophenyl)pyridine **10-5** to form **10-6**. The exchange of cycloiridated ligands is accompanied by the C–F bond cleavage. The exchange occurs under rather harsh conditions and needs a base as opposed to acids, which normally initiate the exchange of cyclometalated ligands. Thus, the formation of **10-6** results from a regioselective hydrodefluorination.

**Figure 10:**
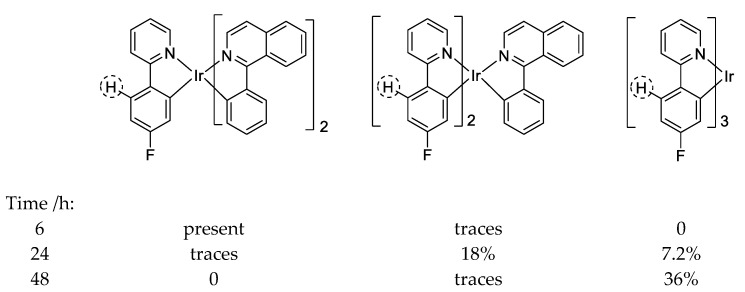
Dynamics of the interaction between **10-4** and **10-5** at 210 °C in glycerol in the presence of K_2_CO_3_.

Though the exchange of cyclometalated ligands commonly involves a C–H bond activation, this transformation occurs through the C–F bond cleavage in the incoming ligand because cycloiridation involving C–F bond dissociation is known [106]. The kinetic data shown in Figure 10 indicate that no cyclometalated complex with intact **10-5** is formed, suggesting the C–F bond cleavage as a key step prior to the C–Ir bond formation.

## 11. Summary and Conclusions

The exchange of cyclometalated ligands is a small chapter in volumes describing cyclometalation, cyclometalated compounds, their reactions, and applications. The exchange is a simple synthetic routine for the preparation of cyclometalated compounds in many instances. It is particularly attractive for the preparation of cyclometalated palladium(II) complexes and pincer platinum(II) and ruthenium(II) derivatives. All exchange processes could be classified into two large groups, viz. (i) exchange in the presence of acidic solvent, co-solvent or additive and (ii) exchange without acidic ingredients. The reactions of the first group are mechanistically more transparent; they involve electrophilic metal centers such as palladium(II), rhodium(III) and sometime platinum(II). Carbon-hydrogen bonds are cleaved by electrophilic metal centers, but carbon-metal bonds undergo protonolysis, i.e., are also cleaved electrophilically. These features point to dissociative mechanisms of the exchange. Here, C–H bond cleavage occurs after C–M bond protonolysis. The exchange of cyclometalated ligands which does not require acidic additives is obviously more delicate, since hydrogen may move intramolecularly from an incoming to leaving ligand as a proton, hydride or even a caged radical. Therefore, associative mechanisms appear, here, more likely because a metal center should bring together both leaving and incoming ligands for creating an optimal pathway for the migration of hydrogen from the latter to the former.

## Figures and Tables

**Table 1:** Synthetically relevant examples of the exchange of cyclopalladated ligands for preparation of selected *N*- and *S*-palladacyles.

Entry	Complex	Ligand	Product	Yield	Reference
1	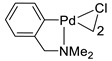 **3-1**	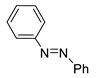	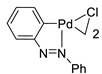	67%	[8]
2	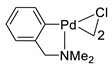	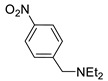	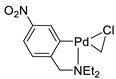	46%	[8]
3	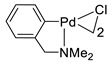	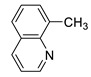	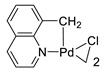	64%	[8]
4	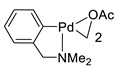 **3-2**	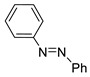	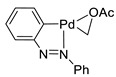	83	[9]
5	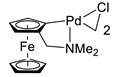 **3-3**	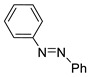	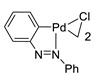	68%	[8]
6	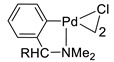 **3-4**	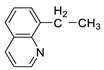	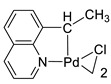	79% (R = H) 55% (R = Me*^R^*) 52% (R = Me*^S^*)	[10]
7	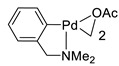	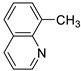	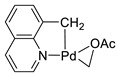	95	[9]
8	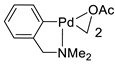	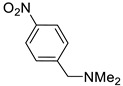	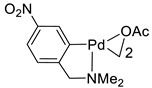	38	[9]
9	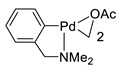	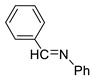	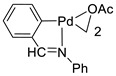	80	[9]
10	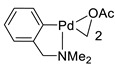	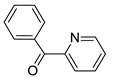	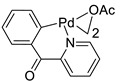	93	[9]
11	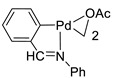	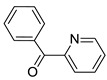	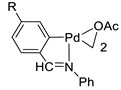 **3-5**	60% (R = Cl) 70% (R = NO_2_)	[11]
12	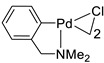	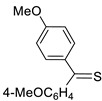	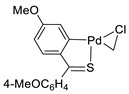	92%	[12]
13	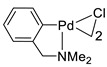	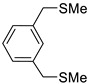	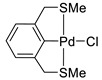	97%	[12]
14	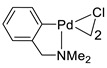	*N,N,N’,N’*-tetramethyl-thiourea	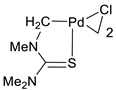	82%	[12]
15	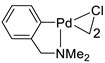	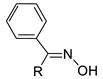	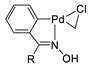 **3-6**	R = H (81%), Me (92%), *^n^*Pr (95%), Ph (86%)	[13,14]
16	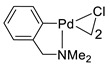	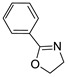	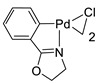	90%	[15]
17	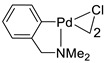	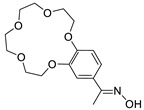	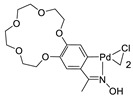 **3-7**	72%	[16]

## References

[1] Dehand J., Pfeffer M. (1976). Cyclometallated compounds. Coord. Chem. Rev..

[2] Ryabov A.D. (1990). Mechanisms of intramolecular activation of carbon-hydrogen bonds in transition-metal complexes. Chem. Rev..

[3] Yatsimirsky A.K., Ryabov A.D., Berezin I.V. (1979). Kinetics and mechanism of oxidative olefin arylation by palladium(II). J. Mol. Catal..

[4] Ryabov A.D., Yatsimirsky A.K. (1980). Reaction of ortho-palladated dimethylbenzylamine with styrene: Unexpected salt effect. Tetrahedron Lett..

[5] Ryabov A.D. (1985). Cyclopalladated Complexes in Organic Synthesis. Synthesis.

[6] Ryabov A.D., Sakodinskaya I.K., Yatsimirsky A.K. (1983). Kinetics and mechanism of vinylation of ortho-palladated N,N-dialkylbenzylamines by para-substituted styrenes. J. Chem. Soc. Perkin Trans..

[7] Ryabov A.D., Polyakov V.A., Yatsimirsky A.K. (1983). Reaction paths in the cyclopalladated N,N-dialkylbenzylamine-substituted styrene system in acetic acid as solvent. The structure of palladated 2-dialkylaminomethylstilbenes. J. Chem. Soc. Perkin Trans..

[8] Ryabov A.D., Kazankov G.M. (1984). The exchange of cyclometalated ligands. II. Attempts to prepare six-membered palladocycles via the ligand exchange reaction. J. Organomet. Chem..

[9] Ryabov D.A., Kazankov G.M. (1986). Exchange of cyclopalladated ligands in palladium acetato dimeric complexes. Koord. Khim..

[10] Ryabov D.A., Kazankov G.M. (1988). Exchange of cyclopalladated ligands involving optically active complexes. Vestn. Mosk. Univ. Ser. 2 Khim..

[11] Granell J., Sainz D., Sales J., Solans X., Font-Altaba M. (1986). Ligand exchange reactions in cyclopalladated complexes of benzylideneanilines. Crystal structure of [Pd(C_6_H_4_CH[double bond, length half m-dash]NC_6_H_5_)Br(PPh_3_)_2_]. J. Chem. Soc. Dalton Trans..

[12] Dupont J., Beydoun N., Pfeffer M. (1989). Reactions of Cyclopalladated Compounds. Part 21. Various Examples of Sulfur-Assisted Intramolecular Palladation of Aryl and Alkyl Groups. J. Chem. Soc. Dalton Trans..

[13] Ryabov A.D., Kazankov G.M., Yatsimirskii A.K., Kuz’Mina L.G., Burtseva O.Y., Dvortsova N.V., Polyakov V.A. (1992). Synthesis by ligand exchange, structural characterization, and aqueous chemistry of ortho-palladated oximes. Inorg. Chem..

[14] Selvakumar K., Vancheesan S., Varghese B. (1997). Synthesis and characterization of cyclopalladated complexes of oximes by ligand-exchange method. Polyhedron.

[15] Smoliakova I.P., Keuseman K.J., Haagenson D.C., Wellmann D.M., Colligan P.B., Kataeva N.A., Churakov A.V., Kuz’Mina L.G., Dunina V.V. (2000). Direct ortho-palladation of 2-phenyl-2-oxazoline. J. Organomet. Chem..

[16] Bezsoudnova E.Y., Ryabov A.D. (2001). Water-soluble cyclopalladated aryl oxime: A potent ‘green’ catalyst. J. Organomet. Chem..

[17] Kazankov M.G., Polyakov V.A., Ryabov A.D., Yatsimirskii A.K. (1990). Synthesis of o-palladated aryl oximes via the exchange of cyclopalladated ligands. Metalloorg. Khim..

[18] Nájera C. (2016). Oxime-Derived Palladacycles: Applications in Catalysis. ChemCatChem.

[19] Yatsimirsky A.K., Kazankov G.M., Ryabov A.D. (2010). Ester Hydrolysis Catalyzed by ortho-Palladated Aryl Oximes. J. Chem. Soc. Perkin Trans..

[20] Ceder R., Gómez M., Sales J. (1989). Ligand exchange reactions of N-donor ligands in cyclopalladated complexes. J. Organomet. Chem..

[21] Albert J., Ceder R.M., Gomez M., Granell J., Sales J. (1992). Cyclopalladation of N-mesitylbenzylideneamines. Aromatic versus aliphatic carbon-hydrogen bond activation. Organometallics.

[22] Gomez M., Granell J., Martinez M. (2000). Mechanisms of cyclopalladation reactions in acetic acid. Not so simple one-pot processes. Eur. J. Inorg. Chem..

[23] Albert J., Granell J., Durán-Mota J.A., Lozano A., Luque A., Mate A., Quirante J., Khosa M.K., Calvis C., Messeguer R. (2017). Endo and exo cyclopalladated (E)-N-([1,1’-biphenyl]-2-yl)-1-mesitylmethanimines: Anticancer, antibacterial and antioxidant activities. J. Organomet. Chem..

[24] Cocco F., Zucca A., Stoccoro S., Serratrice M., Guerri A., Cinellu M.A. (2014). Synthesis and Characterization of Palladium(II) and Platinum(II) Adducts and Cyclometalated Complexes of 6,6′-Dimethoxy-2,2′-bipyridine: C(sp3)–H and C(sp2)–H Bond Activations. Organometallics.

[25] Lamb J.R., Stepanova V.A., Smoliakova I.P. (2013). Transcyclopalladation on silica gel. Polyhedron.

[26] Ryabov A.D., Eliseev A.V., Sergeyenko E.S., Usatov A.V., Zakharkin L.I., Kalinin V.N. (1989). Cyclopalladated o-carboranes. Palladium transfer from cyclopalladated N,N-dimethylbenzylamines, 1C-diphenylphosphino-o-carborane and η3-allyls in carboxylic acid solvents. Polyhedron.

[27] Hiraki K., Fuchita Y., Uchiyama T. (1983). Cyclopalladation of benzyldiphenylphosphine by palladium(II) acetate. Inorganica Chim. Acta.

[28] Usyatinskii Y.A., Ryabov A.D., Bregadze V.I., Shcherbina T.M., Godovikov N.N. (1982). Reaction of some carboranes and their carbon and boron thallated derivatives with palladium(II) acetate. Izv. Akad. Nauk SSSR Ser. Khim..

[29] Sokolov V.I. (1979). Introduction to Theoretical Stereochemistry.

[30] Sokolov V.I. (1983). Asymmetric cyclopalladation as a tool of enantioselective synthesis. Pure Appl. Chem..

[31] Sokolov V. (1995). Optically active organometallic compounds (a personal account from the inside). J. Organomet. Chem..

[32] Dunina V.V. (2011). Chiral Cyclopalladated Compounds: New Structures, Methodologies and Applications. A Personal Account. Curr. Org. Chem..

[33] Sokolov V., Troitskaya L., Reutov O. (1979). Asymmetric cyclopalladation of (dimethylaminomethyl)ferrocene. J. Organomet. Chem..

[34] Dunina V.V., Razmyslova E.D., Gorunova O.N., Livantsov M.V., Grishin Y.K. (2003). Asymmetric exchange of cyclopalladated ligands with a high level of asymmetric induction: A new route to optically active phosphapalladacycles. Tetrahedron: Asymmetry.

[35] Dunina V.V., Turubanova E.I., Livantsov M.V., Lyssenko K.A., Grishin Y.K. (2008). New approach to chiral phosphapalladacycles: First optically active phosphinite PC-palladacycle with non-metallocenic planar chirality. Tetrahedron: Asymmetry.

[36] Ryabov A.D. (1987). Cyclopalladium ligand exchange. Equilibria, kinetics, and mechanism. Zh. Obshch. Khim..

[37] Ryabov D.A., Sakodinskaya I.K., Yatsimirsky A.K. (1985). Kinetics and mechanism of ortho-palladation of ring-substituted N,N-dimethylbenzylamines. J. Chem. Soc. Dalton Trans..

[38] Ryabov A.D. (1987). Thermodynamics, kinetics, and mechanism of exchange of cyclopalladated ligands. Inorg. Chem..

[39] Ryabov D.A., Kuz’mina L., Polyakov V.A., Kazankov G.M., Ryabova E.S., Pfeffer M., van Eldik R. (1995). Kinetics and mechanism of halogen-bridge cleavage in dimethylaminomethylphenyl-C1,N pallada- and platinacycles by pyridines. Pressure effects, and crystal structures of the N,N-cis reaction product, its N,N-trans orthometallated analog and a dimer of similar reactivity. J. Chem. Soc. Dalton Trans..

[40] Ryabov A.D. (1984). Lithium chloride-induced dissociation of cyclopalladated ligands from chloro(ligand-C,N)triphenylphosphinepalladium(II) complexes in acetic acid solvent. J. Organomet. Chem..

[41] Ryabov A., Yatsimirsky A.K., Abicht H.-P. (1987). Protolytic stability of palladocycles. Polyhedron.

[42] Yagyu T., Aizawa S.-I., Funahashi S. (1996). Kinetics and Mechanism of Cyclopalladation for [Pd(S)(Bn2Medptn)](BF4)2(S = Solvent, Bn2Medptn = N,N″-Dibenzyl-N′-methyl-3,3′-diaminodipropylamine) and Its Derivatives. Chem. Lett..

[43] Gomez M.J., Granell J., Martinez M. (1998). Solution behavior, kinetics and mechanism of the acid-catalyzed cyclopalladation of imines. J. Chem. Soc. Dalton Trans..

[44] Yagyu T., Aizawa S.-I., Funahashi S. (1998). Mechanistic Studies on Cyclopalladation of the Solvated Palladium(II) Complexes withN-Benzyl Triamine Ligands in Various Solvents. Crystal Structures of [Pd(Sol)(Bn2Medptn)](BF4)2(Sol = Acetonitrile and N,N-Dimethylformamide; Bn2Medptn = N,N′-Dibenzyl-4-methyl-4-azaheptane-1,7-diamine) and [Pd(H–1Bn2Medptn-C,N,N′,N″)]CF3SO3. Bull. Chem. Soc. Jpn..

[45] Campora J., Lopez J.A., Palma P., Valerga P., Spillner E., Carmona E. (1999). Cleavage of palladium metallacycles by acids: A probe for the study of the cyclometalation reaction. Angew. Chem. Int. Ed..

[46] O’Keefe B.J., Steel P.J. (1999). Cyclometallated compounds XIV. Kinetic versus thermodynamic control in the reversible cyclopalladation of 2-(2-naphthyloxy)pyridine. Inorg. Chem. Commun..

[47] Rogge T., Oliveira J.C.A., Kuniyil R., Hu L., Ackermann L. (2020). Reactivity-Controlling Factors in Carboxylate-Assisted C–H Activation under 4d and 3d Transition Metal Catalysis. ACS Catal..

[48] Lapointe D., Fagnou K. (2010). Overview of the Mechanistic Work on the Concerted Metallation–Deprotonation Pathway. Chem. Lett..

[49] Alharis R.A., McMullin C.L., Davies D.L., Singh K., MacGregor S.A. (2019). The Importance of Kinetic and Thermodynamic Control when Assessing Mechanisms of Carboxylate-Assisted C–H Activation. J. Am. Chem. Soc..

[50] Davies D.L., Donald A.S.M.A., MacGregor S.A. (2005). Computational Study of the Mechanism of Cyclometalation by Palladium Acetate. J. Am. Chem. Soc..

[51] Nonoyama M., Nakajima K., Kita M. (1995). Cyclometallation of N,N-dimethyl-2-bromothiobenzamide and some related thioamides with palladium(0) and palladium(II). Polyhedron.

[52] Zucca A., Cinellu M.A., Pinna M.V., Stoccoro S., Minghetti G., Manassero M., Sansoni M. (2000). Cyclopalladation of 6-Substituted-2,2‘-bipyridines. Metalation of Unactivated Methyl Groups vs Aromatic C–H Activation. Organometallics.

[53] Cope A.C., Friedrich E.C. (2005). Electrophilic aromatic substitution reactions by platinum(II) and palladium(II) chlorides on N,N-dimethylbenzylamines. J. Am. Chem. Soc..

[54] Fuchita Y., Tsuchiya H. (1993). First ortho-palladation of unsubstituted benzylamine by palladium(II) acetate. Polyhedron.

[55] Fuchita Y., Tsuchiya H., Miyafuji A. (1995). Cyclopalladation of secondary and primary benzylamines. Inorganica Chim. Acta.

[56] Vincente J., Saura-Llamas I., Jones P.G. (1993). Orthometallated primary amines. Part 1. Facile preparation of the first optically active cyclopalladated primary amines. J. Chem. Soc. Dalton Trans..

[57] Kurzeev S.A., Kazankov G.M., Ryabov A.D. (2002). Second- and inverse order pathways in the mechanism of orthopalladation of primary amines. Inorganica Chim. Acta.

[58] Ryabov A.D., Eliseev A.V., Yatsimirsky A.K. (1988). Palladium(II)-catalysed Hydrogen/Deuterium allylic exchange in alkenes: An intermediacy of palladium(IV)?. Appl. Organomet. Chem..

[59] Canty A.J. (1993). The +IV Oxidation State in Organopalladium Chemistry. Recent Advances and Potential Intermediates in Organic Synthesis and Catalysis. Platinum Met. Rev..

[60] Canty A.J., Van Koten G. (1995). Mechanisms of d8 organometallic reactions involving electrophiles and intramolecular assistance by nucleophiles. Accounts Chem. Res..

[61] Anklin C., Pregosin P.S., Wombacher F.J., Ruegg H.J. (1990). Mechanistic aspects of the cyclometalation of quinoline-8-carboxaldehyde with platinum(II). Organometallics.

[62] Pregosin P.S., Wombacher F., Albinati A., Lianza F. (1991). Facile cycloplatination of nitrogen compounds. Crystal structure of the cycloplatinated Schiff’s base tetralone derivative PtCl{(cyclohexyl)N:C(CH2)3C6H3 (CO). J. Organomet. Chem..

[63] Navarro-Ranninger C., López-Solera I., Álvarez-Valdés A., Rodriguez-Ramos J.H., Masaguer J.R., Garcia-Ruano J.L., Solans X. (1993). Cyclometalated complexes of palladium(II) and platinum(II) with N-benzyl- and N-(phenylethyl)-.alpha.-benzoylbenzylideneamine. Delocalization in the cyclometalated ring as a driving force for the orthometalation. Organometallics.

[64] Praefcke K., Bilgin B., Pickardt J., Borowski M. (1994). Liquid-crystalline Compounds. 86. The First Disc-Shaped Dinuclear Platinum Mesogen. Chem. Ber..

[65] Navarro-Ranninger C., López-Solera I., González V.M., Pérez J.M., Alvarez-Valdés A., Martín A., Raithby P.R., Masaguer J.R., Alonso C. (1996). Cyclometalated Complexes of Platinum and Palladium withN-(4-Chlorophenyl)-α-benzoylbenzylideneamine. In Vitro Cytostatic Activity, DNA Modification, and Interstrand Cross-Link Studies. Inorg. Chem..

[66] Ghedini M., Pucci D., Crispini A., Barberio G. (1999). Oxidative Addition to Cyclometalated Azobenzene Platinum(II) Complexes: A Route to Octahedral Liquid Crystalline Materials. Organometallics.

[67] Praefcke K., Bilgin B., Pickardt J., Borowski M. (1999). A novel platinum methylene complex. J. Organomet. Chem..

[68] Gorla F., Venanzi L.M., Albinati A. (1994). Synthesis of (1R,1’S)- and (1S,1’S),(1R,1’R)-bis[1-(diphenylphosphino)ethyl]benzene derivatives and their cyclometalation reactions with platinum(II) compounds. The x-ray crystal structures of [2,6-bis[(diphenylphosphino)methyl]phenyl]chloropalladium(II), [(1R,1’S)-2,6-bis[1-(diphenylphosphino)ethyl]phenyl]chloroplatinum(II), and [(1R,1’R),(1S,1’S)-2,6-bis[1-(diphenylphosphino)ethyl]phenyl]chloroplatinum(II). Organometallics.

[69] Helm L., Merbach A.E. (2005). Inorganic and Bioinorganic Solvent Exchange Mechanisms. Chem. Rev..

[70] Helm L., Merbach A.E. (2019). The Periodic Table and Kinetics?. Chim. Int. J. Chem..

[71] Schmuelling M., Ryabov A.D., van Eldik R. (1994). To what extent can the Pt-C bond of a metallacycle labilize the trans position? A temperature- and pressure-dependent mechanistic study. J. Chem. Soc. Dalton Trans..

[72] Ryabov A.D., van Eldik R. (1994). A simple access to homo- and heteroleptic platinum complexes by cycloplatination without the use of organolithium compounds. Angew. Chem. Int. Ed. Engl..

[73] Poveda D., Vivancos Á., Bautista D., González-Herrero P. (2020). Visible light driven generation and alkyne insertion reactions of stable bis-cyclometalated Pt(iv) hydrides. Chem. Sci..

[74] Vivancos Á., Bautista D., González-Herrero P. (2019). Luminescent Platinum(IV) Complexes Bearing Cyclometalated 1,2,3-Triazolylidene and Bi- or Terdentate 2,6-Diarylpyridine Ligands. Chem. Eur. J..

[75] Shaw P.A., Phillips J.M., Clarkson G.J., Rourke J.P. (2016). Trapping five-coordinate platinum(iv) intermediates. Dalton Trans..

[76] Shaw P.A., Phillips J.M., Newman C.P., Clarkson G.J., Rourke J.P. (2015). Intramolecular transcyclometallation: The exchange of an aryl–Pt bond for an alkyl–Pt bond via an agostic intermediate. Chem. Commun..

[77] Shaw P.A., Clarkson G.J., Rourke J.P. (2017). Oxidation of an o-tolyl phosphine complex of platinum: C-H activation and transcyclometallation. J. Organomet. Chem..

[78] Shaw P.A., Clarkson G.J., Rourke J.P. (2017). Reversible C–C bond formation at a triply cyclometallated platinum(iv) centre. Chem. Sci..

[79] Albrecht M., Dani P., Lutz M., Spek A.L., Van Koten G. (2000). Transcyclometalation Processes with Late Transition Metals: Caryl–H Bond Activation via Noncovalent C–H·Interactions. J. Am. Chem. Soc..

[80] Albrecht M., James S.L., Veldman N., Spek A.L., Van Koten G. (2001). Transmetalation reactions with nitrogen-containing “pincer”-class ligands on platinum(II) centers. Can. J. Chem..

[81] Dijkstra H.P., Albrecht M., Medici S., van Klink G.P.M., van Koten G. (2002). Hexakis(PCP-platinum and -ruthenium) complexes by the transcyclometalation reaction and their use in catalysis. Adv. Synth. Catal..

[82] Dijkstra H.P., Albrecht M., van Koten G. (2002). Transcyclometalation, a versatile methodology for multiple metal-carbon bond formation with multisite ligands. Chem. Commun..

[83] Van Klink G.P.M., Dani P., van Koten G. (2002). Bis(ortho-)chelated monoanionic bisphosphinoaryl ruthenium(II) complexes: Synthesis, characterization and reactivity. Chin. J. Chem..

[84] Osakada K. (2003). Transmetalation. Curr. Methods Inorg. Chem..

[85] Estrada-Montaño A.S., Gries A., Oviedo-Fortino J.A., Torres-Gutierrez C., Grain-Hayton A., Marcial-Hernández R., Shen L.Q., Ryabov A.D., Gaiddon C., Le Lagadec R. (2020). Dibromine Promoted Transmetalation of an Organomercurial by Fe(CO)5: Synthesis, Properties, and Cytotoxicity of Bis(2-C6H4-2′-py-κC,N)dicarbonyliron(II). Organometallics.

[86] Cerón-Camacho R., Hernández S., Le Lagadec R., Ryabov A.D. (2011). Cyclometalated [Os(C–N)_x_(N–N)_3−x_]^m+^ mimetics of tris(2,2′-bipyridine)osmium(ii): Covering a 2 V potential range by known (x = 0, 1) and new (x = 2, 3) species (C–N = o-2-phenylpyridinato). Chem. Commun..

[87] Le Lagadec R., Alexandrova L., Estevez H., Pfeffer M., Laurinavičius V., Razumienė J., Ryabov A.D. (2006). Bis-Ruthena(III)cycles [Ru(C∩N)2(N∩N)]PF6 as Low-Potential Mediators for PQQ Alcohol Dehydrogenase (C∩N = 2-phenylpyridinato or 4-(2-tolyl)pyridinato, N∩N = bpy or phen). Eur. J. Inorg. Chem..

[88] Ryabov A.D., Sukharev V.S., Alexandrova L., Le Lagadec R., Pfeffer M. (2003). Low-Potential Cyclometalated Osmium(II) Mediators of Glucose Oxidase. Inorg. Chem..

[89] Saavedra-Díaz O., Cerón-Camacho R., Hernández S., Ryabov A.D., Le Lagadec R. (2008). Denial of Tris(C,N-cyclometalated) Ruthenacycle: Nine-Membered η6-N,N-transor η2-N,N-cisRuIIChelates of 2,2′-Bis(2-pyridinyl)-1,1′-biphenyl. Eur. J. Inorg. Chem..

[90] Jolliet P., Gianini M., Von Zelewsky A., Bernardinelli G., Stoeckli-Evans H. (1996). Cyclometalated Complexes of Palladium(II) and Platinum(II): Cis-Configured Homoleptic and Heteroleptic Compounds with Aromatic C⌒N Ligands. Inorg. Chem..

[91] Sanna G., Pilo M.I., Spano N., Minghetti G., Cinellu M.A., Zucca A., Seeber R. (2001). Electrochemical behaviour of cyclometallated gold(III) complexes. Evidence of transcyclometallation in the fate of electroreduced species. J. Organomet. Chem..

[92] Aydin J., Selander N., Szabó K.J. (2006). Strategies for fine-tuning the catalytic activity of pincer-complexes. Tetrahedron Lett..

[93] Dani P., Albrecht M., Van Klink G.P.M., Van Koten G. (2000). Transcyclometalation: A Novel Route to (Chiral) Bis-Ortho-Chelated Bisphosphinoaryl Ruthenium(II) Complexes. Organometallics.

[94] Gagliardo M., Chase P.A., Lutz M., Spek A.L., Hartl F., Havenith R.W.A., Van Klink G.P.M., Van Koten G. (2005). A Ruthenium(II) Complex Stabilized by a Highly Fluorinated PCP Pincer Ligand. Organometallics.

[95] Gagliardo M., Dijkstra H.P., Coppo P., De Cola L., Lutz M., Spek A.L., Van Klink G.P.M., Van Koten G. (2004). Synthesis, Crystal Structure, and Redox and Photophysical Properties of Novel Bisphosphinoaryl RuII-Terpyridine Complexes. Organometallics.

[96] Medici S., Gagliardo M., Williams S.B., Chase P.A., Gladiali S., Lutz M., Spek A.L., Van Klink G.P.M., Van Koten G. (2005). Novel P-Stereogenic PCP Pincer-Aryl Ruthenium(II) Complexes and Their Use in the Asymmetric Hydrogen Transfer Reaction of Acetophenone. Helv. Chim. Acta.

[97] Gagliardo M., Perelaer J., Hartl F., Van Klink G.P.M., Van Koten G. (2007). Mono- and Heterodimetallic FeII and RuII Complexes Based on a Novel Heteroditopic 4′-{Bis(phosphanyl)aryl}-2,2′:6′,2″-terpyridine Ligand. Eur. J. Inorg. Chem..

[98] Dani P., Toorneman M.A.M., Van Klink G.P.M., Van Koten G. (2000). Complexes of Bis-ortho-cyclometalated Bisphosphinoaryl Ruthenium(II) Cations with Neutral Meta-bisphosphinoarene Ligands Containing an Agostic C–H·Ru Interaction. Organometallics.

[99] Djukic J.-P., Maisse A., Pfeffer M. (1998). Cyclomanganated (η6-arene)tricarbonylchromium complexes: Synthesis and reactivity. J. Organomet. Chem..

[100] Djukic J.-P., Maisse A., Pfeffer M., De Cian A., Fischer J. (1997). Synthesis and Reactivity of New Cyclomanganated (η6-Arene)tricarbonylchromium Complexes. Organometallics.

[101] Bruce M., Liddell M., Snow M.R., Tiekink E.R. (1988). Cyclometallation Reactions. XXI. Some Chemistry of Binuclear Manganese Complexes Derived From Azobenzene: X-Ray Crystal-Structures of {Mn (CO)_4_}_2_(μ-C_6_H_4_N=NC_6_H_4_) and {Mn(CO)_4_}{Mn(CO)_3_[P(OPh)_3_]}(μ-C_6_H_4_N=NC_6_H_4_). Aust. J. Chem..

[102] Robinson N.P., Main L., Nicholson B.K. (1992). Orthomanganated arenes in synthesis VIII. Mono- and dicyclomanganation of diacetyl benzenes. J. Organomet. Chem..

[103] Vanderweide A.I., Brennessel W.W., Jones W.D. (2019). Reversible Concerted Metalation–Deprotonation C–H Bond Activation by [Cp*RhCl_2_]_2_. J. Org. Chem..

[104] Donnelly K.F., Lalrempuia R., Müller-Bunz H., Clot E., Albrecht M. (2015). Controlling the Selectivity of C–H Activation in Pyridinium Triazolylidene Iridium Complexes: Mechanistic Details and Influence of Remote Substituents. Organometallics.

[105] Lepeltier M., Dumur F., Marrot J., Contal E., Bertin D., Gigmes D., Mayer C.R. (2013). Unprecedented combination of regioselective hydrodefluorination and ligand exchange reaction during the syntheses of tris-cyclometalated iridium(iii) complexes. Dalton Trans..

[106] Perera S.D., Shaw B.L., Thornton-Pett M. (1995). Cyclometallation of a pentafluorobenzaldehyde azine phosphine via a C-F bond fission by irridium(I): Crystal structure of [IrCl_2_(CO){PPh_2_CH_2_C(but):NN:CH(C_6_F_4_)}]. Inorganica Chim. Acta.

